# Dual Action Nitric Oxide-Releasing Polydimethylsiloxane Sponge: Preventing Infection in Needleless Connectors

**DOI:** 10.1021/acsabm.5c01100

**Published:** 2025-09-12

**Authors:** Adam Brooks Goodman, Manjyot Kaur Chug, Natalie Crutchfield, Mark Garren, Hitesh Handa, Elizabeth J. Brisbois

**Affiliations:** † School of Chemical, Materials, and Biomedical Engineering, College of Engineering, 1355 University of Georgia, Athens, Georgia 30602, United States; ‡ Department of Pharmaceutical and Biomedical Sciences, College of Pharmacy, University of Georgia, Athens, Georgia 30602, United States

**Keywords:** nitric oxide, isopropanol, antimicrobial, polydimethylsiloxane sponge, disinfecting caps

## Abstract

Catheter-related bloodstream infections (CRBSIs) are a prevalent concern, often resulting from suboptimal disinfection practices of needleless connectors. Although alcohol-based disinfectants have demonstrated efficacy, there is growing concern about developing microbial resistance. Similar to antibiotics in recent decades, microbes have the potential to develop resistance to these alcohol-based therapies. Therefore, this study delves into the antimicrobial potential of nitric oxide (NO), an endogenous gas molecule with broad-spectrum antimicrobial properties, in combination with the widely used disinfectant 70% isopropanol (IPA). Due to its short half-life, NO presents minimal risk of microbial resistance development. By incorporating *S*-nitroso-*N*-acetylpenicillamine (SNAP), a synthetic NO donor, into hydrophilic-modified polydimethylsiloxane (PDMS-PEO) sponges using 70% IPA, the sponge functions as an antimicrobial reservoir, effectively sterilizing the hub region of needleless connectors. Formulation-dependent effects on sponge porosity were observed, affecting compressive strength, absorption capacity, SNAP retention, and NO release kinetics. Up to 30% variation in sponge porosity coincided with a tunable compressive modulus, increased absorption capacity of 70% IPA, and enhanced SNAP loading after 15 min. These properties enable significantly greater SNAP release within 30 min. Zone of inhibition demonstrated higher porosity leads to more significant inhibition of *Escherichia coli*, *Pseudomonas aeruginosa*, *Staphylococcus aureus*, *Staphylococcus epidermidis*, and *Candida albicans*. The disinfection of needleless connectors demonstrated a 2.91-, 7.04-, 2.02-, 3.21-, and 5.65-log reduction in *E. coli*, *P. aeruginosa*, *S. aureus*, *S. epidermidis*, and *C. albicans* viability after 30 min. These findings highlight the potential of this approach for efficient microbial decontamination in healthcare settings while offering adaptability for diverse biomedical applications.

## Graphical abstract

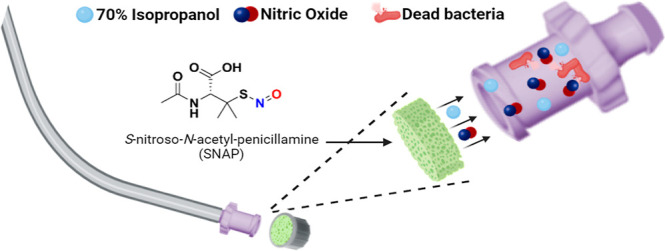

## 1 Introduction

Hospital-acquired infections (HAIs) persist as a significant and ongoing challenge in modern society, despite continuous advancements in biomedical engineering aimed at their prevention. Tragically, HAIs rank as the fourth leading cause of death in the U.S., caused by various virulent microbes such as *Staphylococcus aureus*, *Escherichia coli*, and *Candida albicans* *.* ^1^ These morbid infections primarily stem from the use of medical devices, particularly catheters. In the United States, more than 30,000 catheter-related bloodstream infections (CRBSIs) occur annually, resulting in a financial burden of over $1 billion in medical costs for patients and a higher risk of mortality.^2,3^

One of the commonly used intravascular catheter accessories employed by healthcare professionals is needleless connectors. Unfortunately, these connectors pose a significant contamination risk after use for various reasons, including multiple personnel handling the area, insufficient training and disinfection practices, and neglection of adequate hand hygiene.^4^ This post-use contamination often results in biofilm formation, which creates a formidable barrier to effective microbe eradication.^4–6^ Microbes can thrive and establish colonies within the intraluminal pathway of catheters via contamination of the hub region.^7^ While antibiotic lock solutions have traditionally been the go-to approach for treating microbial infections and eliminating formed biofilms, the effectiveness of antibiotics has been diminishing over the decades due to the development of microbial resistance resulting from improper use in healthcare settings. This disrupts the balance of beneficial microbes that naturally combat infections, allowing drug-resistant microbes to proliferate unchecked.^8^

In response to the decreasing effectiveness of antibiotics, innovative medical devices have emerged to address microbial infections associated with catheters, offering an alternative approach in line with stewardship practices. Among these innovations, antiseptic barrier caps have gained attention for their capacity to disinfect needleless connectors by using sponge materials soaked in disinfecting agents, such as 70% isopropyl alcohol (IPA). The antibacterial properties of 70% IPA have been well-documented and extensively studied,^9–12^ demonstrating exceptional efficacy in eradicating a wide range of microbes.^13^ However, much like antibiotics, improper use of alcohol-based therapies can lead to microbial resistance.^14,15^ Moreover, 70% IPA may not provide comprehensive protection against all pathogens, including antibiotic-resistant species. Certain microorganisms, such as specific bacterial and fungal spores, exhibit heightened resistance to alcohol-based disinfectants like IPA.^16^ Given the susceptibility of needleless connectors to diverse microbial threats in healthcare settings, it becomes evident that supplementary disinfection methods or antimicrobial agents are essential to achieve comprehensive protection.

In this context, nitric oxide (NO), a small molecule naturally produced by various cell types within the human body, plays a crucial role. Nitric oxide, when present at higher concentrations, transforms into a potent antimicrobial agent. As a gaseous molecule, NO possesses the remarkable ability to directly penetrate microbial membranes, even infiltrating established biofilms.^17,18^ It effectively denatures lipids, proteins, and DNA of microorganisms by generating reactive nitrogen species (RNS) and reactive oxygen species (ROS) through side reactions.^19^ Notably, the incredibly short half-life of NO makes it exceedingly challenging for microbes to develop resistance to its antimicrobial effects.^20,21^ In this study, *S*-nitroso-*N*-acetylpenicillamine (SNAP), a synthetic *S*-nitrosothiol (RSNO), was used as an NO donating compound for its ability to release NO in the presence of heat, light, or metal ions.^22^ As a NO-donor, SNAP has demonstrated substantial antimicrobial efficacy against Gram-positive and Gram-negative microbes and various fungal species, leading to its application in diverse biomedical settings, including vascular catheters, urinary catheters, and coatings for medical devices.^23^ Previous research using RSNOs has demonstrated the ability to integrate NO donors into collagen-based sponges for wound healing. Despite the proven effectiveness of these materials in promoting wound healing, there is still a lack of comprehensive understanding regarding the antimicrobial capabilities of the NO-releasing sponge.^24^

This study explores the development of a NO-releasing sponge integrated within needleless connectors to prevent microbial contamination and CRBSIs. Polydimethylsiloxane (PDMS) sponges were fabricated using a salt template removal method. This involved adding sodium chloride (NaCl) particles into the PDMS polymer, which were subsequently dissolved in hot water after the PDMS had cured. To impart hydrophilic properties into the sponge, an amphiphilic surfactant (poly­(dimethylsiloxane-*b*-ethylene oxide), methyl terminated) (PDMS-*b-*PEO) was blended into the fabrication process.^25,26^ The porosity of the sponge material was modified by altering the concentration of NaCl added to the pre-crosslinked solution, generating sponges with various porosities. To incorporate NO-releasing properties into the PDMS sponge, the NO donor SNAP was infused with 70% IPA as the SNAP-carrier phase, representing a promising avenue within porous materials and their practical utility. Scanning electron microscopy (SEM) techniques were employed to investigate the sponges’ structural changes resulting from the surfactant’s addition. To further characterize the sponges, the compressive strength was evaluated by measuring the force required to compress the sponge up to 50% strain uniaxially. The absorption capacity of 70% IPA, maximum SNAP incorporation, and NO release kinetics were analyzed using a UV–vis and by observing the difference in sponge weight over time, among other spectroscopic measurement techniques. A zone of inhibition study was employed to gain insights into the relationship between porosity and the antimicrobial activity of the SNAP-IPA-loaded sponges. In addition, the contact-killing capability of the SNAP-IPA-loaded sponges was also explored using a 4 h antimicrobial assay. Subsequently, SNAP-IPA sponges were subjected to a simulated healthcare environment using pre-contaminated needleless connectors to assess the disinfection potential. The potent action of SNAP with 70% IPA presents a highly promising approach for disinfecting medical devices, with minimal potential for developing microbial resistance. The antimicrobial property of this material exhibits significant potential for infection prevention in healthcare settings, addressing a critical need in the field of HAIs.

## 2 Materials and Methods

### 2.1 Materials

Poly­(dimethylsiloxane) (PDMS) base and curing agent (Sylgard 184) were purchased from Ellsworth Adhesives. BD DIFCO yeast mold (YM) broth, BD DIFCO YM agar, concentrated hydrochloric acid (conc. HCl, 12.1 M), crystalline sodium chloride, and ethanol (100%) were purchased from Fisher Scientific, Inc. Poly­(dimethylsiloxane-*b*-ethylene oxide), methyl terminated (PDMS-*b-*PEO) was purchased from Polyscience (Warrington, PA). *S-*Nitroso-*N*-acetylpenicillamine (SNAP, >97%) was purchased from PharmaBlock (Hatfield, PA) with purity verified via its catalytic decomposition and subsequent chemiluminescence-based detection of evolved NO (>90% purity by mol of evolved NO per mol SNAP). Luria–Bertani (LB) broth, LB agar, phosphate-buffered saline (PBS), sodium nitrite (>99.0%), methanol (>99.8%), concentrated sulfuric acid (conc. H_2_SO_4_, 18 M), ethylenediaminetetraacetic acid (EDTA), and isopropanol (IPA) were purchased from Sigma-Aldrich (St. Louis, MO). Polycarbonate, stemless, nonvented, clear male luer caps, and female luer lock-to-barb connectors were purchased from Qosina (Ronkonkoma, NY). Helix Mark silicone tubing (outer diameter 1.96 mm) was purchased from VWR (Radnor, PA). 3 M Tegaderm roll transparent dressing—nonsterile was purchased from 3 M (Maplewood, MN). *S. aureus* (ATCC 6538), *Staphylococcus epidermidis* (*S. epidermidis*, ATCC 35984), *E. coli* ATCC (25922), *Pseudomonas aeruginosa* (*P. aeruginosa*, ATCC 27853), and *C. albicans* (ATCC MYA4441) were obtained from the American Type Culture Collection (Manassas, VA). Phosphate buffered saline (10 mM, pH 7.4), LB media, and YM media were dissolved in water and sterilized in an autoclave at 121 °C, 100 kPa (15 psi) above atmospheric pressure for 30 min before bacterial studies. All chemicals were reagent-grade and used as-is without further purification.

### 2.2 Hydrophilic Polydimethylsiloxane Sponge Fabrication

To impart hydrophilicity to the naturally hydrophobic PDMS material, a controlled amount of PDMS-*b-*PEO was introduced into the PDMS base and curing agent mixture for 2 min. The incorporation of PDMS base into curing agent was initially carried out thoroughly at a ratio of 10:1 for 2 min. Sodium chloride (NaCl) crystals were manually stirred in for an additional 2 min until complete homogenization was achieved. The resulting blend was then compacted into cylindrical glass molds and cured in an oven at 100 °C for 15 h. Once cured, the PDMS sponges, which exhibited varying salt concentrations ranging from 400 to 1200% m/m (referred to as 16–48 g) in relation to the initial PDMS base agent, were carefully removed from the oven. Sponges were then immersed in hot water for at least 8 h to facilitate dissolution and completely remove all NaCl particles, exchanging the water every 4 h. After extraction from the molds, the sponges were dried in a 100 °C oven for 4 h. A rinsing procedure involving ethanol was performed to eliminate any residual salt on the outer surface, followed by thorough drying. This process was additionally performed without the addition of surfactant to fabricate control PDMS sponges with the same salt concentrations.

### 2.3 Porosity of Polydimethylsiloxane Sponge

The effective porosity of hydrophilic sponges was assessed through absorption in water for 24 h at room temperature.^27^ Sponge samples without the surfactant were subjected to the same process. However, due to the hydrophobic nature of PDMS, samples were initially placed in ethanol for 4 h and then placed in 1 mL of fresh water every hour for 4 h before the 24 h absorption period. Values were calculated using the mass per volume density of sponges before and after absorption using eq 1.

1 $$porosity\;\left(\%\right)=\left(1-\frac{\rho }{\rho_{s}}\right)\times 100\%$$

Where ρ and ρ_s_ are the density of the sponge before and after water uptake.

To align with the SNAP loading and in vitro conditions, the porosity of sponges was also calculated using 70% IPA as the swelling medium, following the same procedure and eq 1. This provided a more relevant measurement under the solvent environment used in subsequent experiments. However, water-swelling porosity values were used throughout the study to classify and compare sponge groups, as water-based measurements are more widely accepted for assessing hydrophilic materials. The use of IPA was intended primarily to enhance SNAP solubility and loading efficiency, as well as to leverage its disinfecting properties, rather than to redefine sponge porosity.

### 2.4 Evaluation of Compressive Strength

Uniaxial compression testing was performed using a Mark-10 Series 5 force gauge (Mark-10, Copiague, NY). The sponges were compressed to 50% strain at a rate of 1.3 mm min^–1^. The mechanical testing was completed with cylindrical samples (approximately 8 mm in diameter and 8 mm in height). The diameter and thickness of each sample were measured from at least three different starting points along its length with an accuracy of 0.025 mm. The minimum value of the cross-sectional area, along with the length of each sample, was recorded for further analysis. The stress–strain relationship was analyzed (*n* = 5) to determine the modulus of elasticity.

### 2.5 Scanning Electron Microscopy

To examine the surface morphology and pore size of the various PDMS sponges, microscopy techniques were employed. Samples with an approximate diameter of 6 mm were prepared for imaging by applying a 10 nm gold-palladium coating using a Leica sputter coater (Leica Microsystems). Scanning electron microscopy (SEM, FEI Teneo, FEI Co.) was used to capture high-resolution images of the cross-sectional morphology and porosity of the different sponge formulations. A total of 30 sites were analyzed for each sample formulation (*n* = 1) using Gaussian least squares fitting and ImageJ imaging software (National Institutes of Health, Bethesda, MD) to obtain pore size distributions.

### 2.6 Surface Water Contact Angle of Polydimethylsiloxane Sponges

Hydrophilic modification of the PDMS sponge was assessed by depositing 10 μL droplets of DI water on both the hydrophobic (without PEO) and hydrophilic (with PEO) sponges. Video recordings using an Ossila Contact Angle Goniometer (Sheffield, UK) were used to capture the droplets on the sponge surfaces, and snapshots were extracted to monitor the evolution of droplet morphology over time.

### 2.7 Universal Attenuated Total Reflectance-Fourier Transform Infrared Spectroscopy

A spectrum two spectrometer from PerkinElmer (Greenville, SC) was used to perform FTIR spectroscopic measurements for the sponge samples. Spectral reflectance mode with the UATR accessory was employed, providing a resolution of 4 cm^–1^ within the 4000–650 cm^–1^ range to detect the PEO surfactant added to the sponges. A total of 128 scans were performed. Samples were dried before testing and the 82% porous sponge formulation was used as a representative control. Measurements were carried out for both representative PDMS and PDMS-PEO samples to identify any functionalized differences.

### 2.8 Absorption Capacity of Sponge

Absorption capacities of the hydrophilic sponges were evaluated by measuring the change in mass of the samples after 15, 30, and 60 min in 70% IPA (*n* = 3) using eq 2.

2 $$swelling\;ratio\;\left(\%\right)=\frac{m_{f}-m_{0}}{m_{0}}\times 100\%$$

Where *m*_f_ is the swollen weight after soaking in 70% IPA and *m*_0_ is the initial weight of the sponge.

### 2.9 *S*-Nitroso-*N*-acetylpenicillamine Loading into Sponge

A SNAP solution was prepared by dissolving SNAP in 70% IPA (85 mg mL^–1^) using a vortex. Hydrophilic sponges, measuring 4.5 mm in diameter and 5 mm thick, were submerged in 0.6 mL of the solution within 1.5 mL microcentrifuge tubes. Sponges were added to the solution for 15, 30, or 60 min before being placed in a fume hood covered from light for 24 h to fully evaporate the IPA. The dried SNAP sponges were transferred to 10 mL fresh 70% IPA to extract the SNAP completely. The concentration of SNAP in each sample (*n* = 3) was determined using a UV–vis spectrophotometer (Cary 60, Agilent Technologies). The molar absorptivity of SNAP was determined to be 875 M^–1^ cm^–1^ using a standard curve of SNAP in 70% IPA. A quartz cuvette was used to measure the absorbance of the samples at 340 nm, as this wavelength corresponds to the absorbance maxima of the S–NO group present in SNAP.^28^

### 2.10 Simulated Catheter Delivery of *S*-Nitroso-*N*-acetylpenicillamine

To investigate the release of SNAP in a relevant setting, SNAP-IPA sponges underwent a simulated catheter setup using silicone tubing (outer diameter = 1.96 mm) equipped with a luer connector and cap. Luer connectors were attached to one end of silicone tubing cut to approximately 76.2 mm in length, which was then filled with 10 mM PBS, pH 7.4, with 100 μM EDTA. The opposite end of the tubing was clamped, and SNAP-IPA sponges were carefully placed inside luer caps and securely fastened onto the luer connectors. Sponges were removed from the caps after 0.5, 1, 4, or 24 h and dried in a fume hood with protection from light to ensure the complete removal of IPA. Following the same technique used for SNAP loading (Section 2.9), the dried sponges were subjected to SNAP extraction, and the resulting solution was analyzed for absorbance using a UV–vis spectrophotometer.

For optimal fit within the luer cap, sponges were cut out using a 1/4-in. diameter punchout and trimmed to a thickness of 5 mm. The samples were weighed before and after addition to the SNAP-IPA solution to determine the theoretical amount of SNAP incorporated into the sponges using eq 3.

3 $$SNAP\;wt\;\left(mg\right)=\frac{m_{f}-m_{0}}{\rho }\times C$$

Where *m*_f_ is the swollen weight of the sponge, *m*_0_ is the initial weight of the sponge, ρ is the density of 70% IPA in mg mL^–1^ at room temperature, and *C* is the concentration of SNAP in IPA in mg mL^–1^. Using the concentration of SNAP determined from the absorbance measurements and the values calculated from eq 3, the amount of SNAP remaining in the samples (*n* = 3) was determined. Subsequently, the total amount of SNAP delivered was calculated, expressed as nmol mg^–1^.

### 2.11 Storage Stability of *S*-Nitroso-*N*-acetylpenicillamine-Isopropanol Sponge

The 82% porous SNAP-IPA sponge was used to evaluate the stability of the material over time. After swelling, SNAP-IPA sponges (1/4 in. diameter, 5 mm thickness) were carefully placed into luer caps, which were sealed with a thin piece of aluminum foil and parafilm to minimize IPA evaporation and mimic practical storage conditions. Samples were stored in the dark at room temperature, 4 °C, or −20 °C, and removed after 1, 4, or 7 d. Sponges were dried in a vacuum desiccator, protected from light, to ensure complete IPA evaporation. Following the SNAP extraction method described in Section 2.9, dried sponges were immersed in 2 mL of PBS with EDTA at 37 °C for 24 h. The resulting leachates were analyzed for nitrite production via the Griess assay and for residual SNAP content using UV–vis spectroscopy. Sponges were weighed before and after storage to evaluate changes in mass over time using eq 4.

4 $$SNAP\;wt\;\left(mg\right)=\frac{m_{i}-m_{f}}{m_{i}}\times 100\%$$

Where *m*_i_ is the initial swollen weight of the sponge and *m*_f_ is the final weight after storage.

To assess SNAP stability, leachates were measured at 340 nm using a UV–vis spectrophotometer (Cary 60, Agilent Technologies). The absorbance values were converted to SNAP concentrations using a molar absorptivity of 1058 M^–1^ cm^–1^, determined from a standard curve of SNAP in PBS with EDTA.

In parallel, 50 μL of each leachate sample was reacted with 50 μL of a 40 mg mL^–1^ Griess reagent solution (20 mg mL^–1^ final concentration) in PBS. The resulting azo dye was measured at 540 nm using a plate reader (BioTek Cytation 5 imaging reader). Nitrite concentrations were calculated using a sodium nitrite standard curve (0–5 μg/mL), and the molar absorptivity at 540 nm was determined to be 1.09 × 10^4^ M^–1^ cm^–1^.

### 2.12 Growth of Microbial Cultures

A single isolated colony of *E. coli*, *P. aeruginosa*, *S. aureus*, and *S. epidermidis* was inoculated in LB media and incubated at 37 °C for 15 h at 150 rpm. Over time, the growth of the microbial cultures was assessed by measuring the optical density (OD) of the microbial suspensions at 600 nm using a UV–vis spectrophotometer (Cary 60, Agilent Technologies). A midlog phase of each bacteria type was extracted from the suspensions by centrifuging the cultures at 4400 rpm for 7 min. Suspensions were adjusted to an OD_600_ of 0.1, corresponding to ∼10^7^ colony-forming units (CFUs) mL^–1^ for further analysis. Similarly, *C. albicans* was grown under the same conditions using YM media, with an initial incubation period of 20 h.

### 2.13 Antimicrobial Efficacy via Zone of Inhibition Assay

The antimicrobial efficacy of the NO-releasing sponge was evaluated against *E. coli*, *P. aeruginosa*, *S. aureus*, *S. epidermidis*, and *C. albicans*. A 50 μL sample of adjusted microbial suspension (0.1 OD_600_) of each microbial type was evenly spread onto LB or YM agar plates using a cotton applicator. The plates were allowed to air-dry at room temperature for 5 min. Unmodified, IPA control, and SNAP-IPA sponges (4.5 mm diameter, 5 mm thick) were placed equidistant on the microbial agar plates (*n* = 4). Plates were incubated at 37 °C for 24 h and the diameter of the zone around the sponges was measured to assess the inhibited growth of the microbe. The results from the zone of inhibition study were recorded by measuring the diameter (in cm) of the regions where microbial growth was absent around the respective sponge samples. Findings from the study are presented as the mean diameter ± standard deviation (SD, *n* = 4).

### 2.14 Evaluation of Antimicrobial Efficacy via Contact-Killing Study

To further investigate the antimicrobial activity, 70% IPA and SNAP-IPA sponges were evaluated using a 4 h contact-killing study. For this, unmodified control, 70% IPA control, or SNAP-IPA sponges, were added to 1.5 mL microcentrifuge tubes containing 1 mL of 0.1 OD_600_ microbial suspension (*E. coli*, *S. aureus*, and *C. albicans*). Samples were incubated at 37 °C for 4 h to let the SNAP and IPA act on bacteria and fungi. After 4 h of incubation, samples were discarded from the solutions, and the remaining viable microbes in the suspension were serially diluted and spread on LB agar for *E. coli* and *S. aureus* or YM agar for *C. albicans* using a bacteria spiral plater (Eddy Jet 2, IUL Instruments). Microbial plates were further incubated at 37 °C for 24 h to allow colonies to grow for CFU counting. The viable CFUs from the study were enumerated using an automated bacteria colony counter (Sphere Flash, IUL Instruments). The percentage and log reductions for each microbe were calculated using eqs 5 and 6 and normalized to an average swollen volume after 15 min in 70% IPA.

5 $$bacterial\;reduction\;\left(\%\right)=\frac{{CFU}_{control}-{CFU}_{treatment}}{{CFU}_{control}}\times 100\%$$

6 $$\mathrm{Log}reductionin\;viable\;bacteria={\mathrm{Log}}_{10}\left({CFU}_{control}\right)-{\mathrm{Log}}_{10}\left({CFU}_{treatment}\right)$$

### 2.15 In Situ Disinfection of Needleless Connectors Using Infection Model

To evaluate the microbial disinfection efficacy of the SNAP-IPA sponge, female luer connectors were preinfected with microbes. This configuration was designed to mimic the conditions experienced in clinical environments. For this, *E. coli*, *P. aeruginosa*, *S. aureus*, *S. epidermidis*, and *C. albicans* were grown following a similar methodology as Section 2.11. The inner area of female luer connectors was first exposed to 75 μL of adjusted microbial suspension (0.1 OD_600_) in media for 6 h at 37 °C. After exposure, the media from the connectors was carefully discarded, and connectors were briefly rinsed with 75 μL of sterile PBS to remove any unadhered microbes. The connectors were allowed to dry at room temperature for 10 min before introducing IPA or SNAP-IPA sponges (1/4 in diameter, 5 mm thick) into luer caps, securing them firmly onto the connectors. After an incubation period of 30 min at 37 °C, the caps were removed from the luer connectors, and the connectors were transferred to 15 mL vials containing 3 mL of PBS. Each connector was homogenized and vortexed for 1 min to extract the remaining viable adhered microbes on the connectors. The resulting solutions were diluted and plated onto LB or YM agar plates using a bacteria spiral plater (Eddy Jet 2, IUL Instruments). Microbial plates were then incubated at 37 °C for 24 h to facilitate efficient colony growth for CFU counting using an automated bacteria colony counter (Sphere Flash, IUL Instruments). The percent and log reductions for viable microbes were determined through eqs 5 and 6 and normalized based on the treated suspension volume, respectively. Control connectors were subjected to the same process; however, no disinfecting cap was added to the connectors after the initial microbial exposure.

### 2.16 Statistical Analysis

Data are presented as mean ± standard deviation (SD) with a sample size of *n* ≥ 3, unless specified otherwise. Statistical analyses were performed using Prism 9.1 (GraphPad Software, San Diego, CA). A standard one-way analysis of variance (ANOVA) was used to compare the treatment groups by assessing the average values. Multiple comparisons were conducted to evaluate the differences between the average values of the sample groups. Statistical significance was defined as *p* < 0.05.

## 3 Results and Discussion

### 3.1 Fabrication of Nitric Oxide-Releasing Hydrophilic Polydimethylsiloxane Sponges

The fabrication of PDMS sponges is a well-established and versatile process with a recent history of applications in drug loading and release.^27,29,30^ For this, PDMS, PDMS-*b*-PEO, and NaCl were combined to produce hydrophilic sponges of varying porosities by adjusting the concentration of the porogen (NaCl) following previously published studies (Figure 1A).^26,31^ Fabricated sponges were stored in a sealed environment at room temperature for future use. To impart NO-releasing attributes to the sponges, samples were swollen with a SNAP-IPA solution (85 mg mL^–1^), slightly below the solubility limit (90 mg mL^–1^), to ensure even distribution of SNAP without the potential for SNAP to crash out. Control samples for the experiment consisted of sponges with only 70% IPA solution, without the inclusion of SNAP. All samples were freshly prepared and not subject to storage.

**Figure 1:**
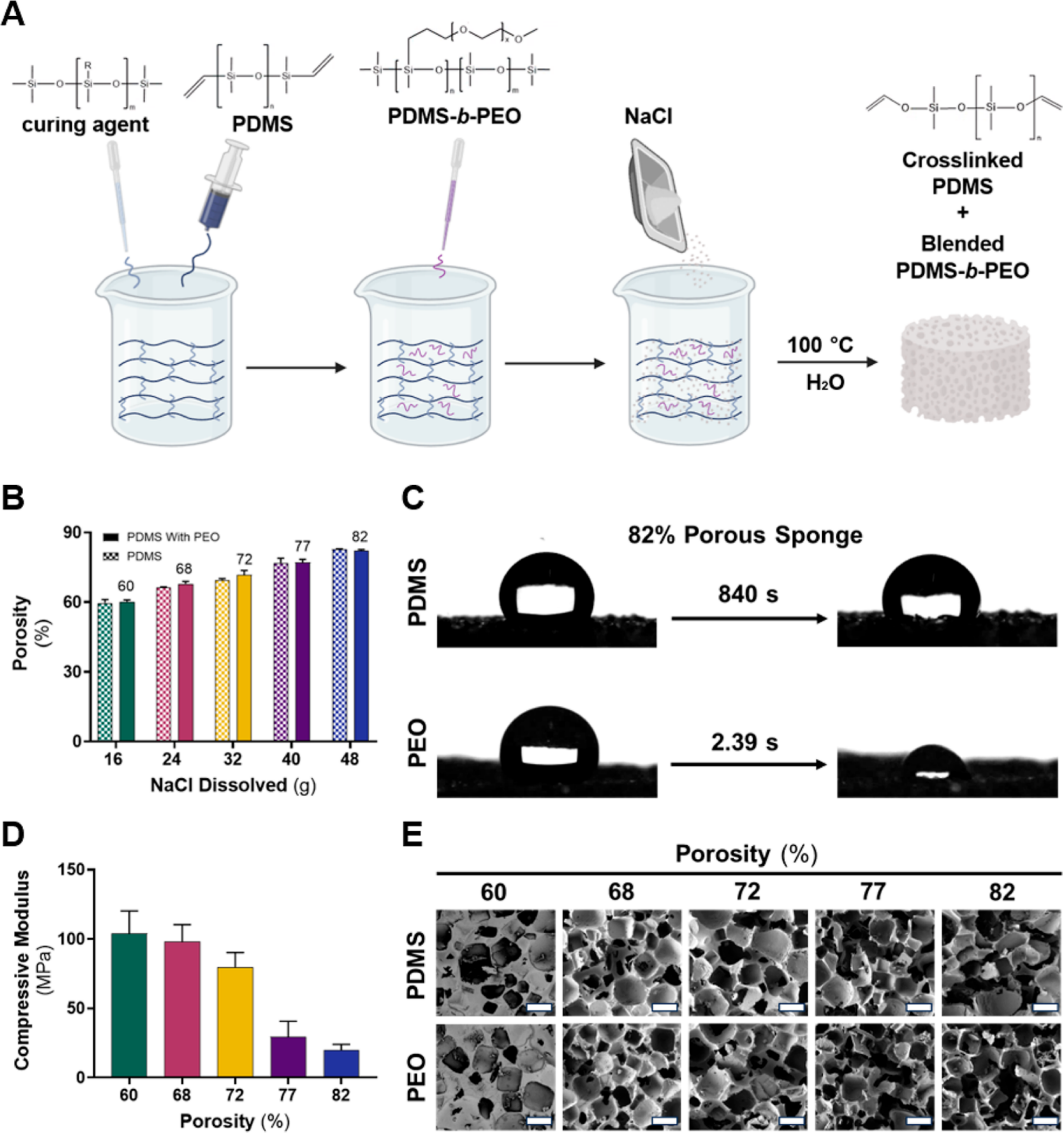
PDMS sponge characterization. (A) Hydrophilic PDMS sponge fabrication. After mixing each component for 2 min, the resulting solution was compacted into glass molds and cured at 100 °C for 15 h. Molds were added to hot water to dissolve the salt template then washed with ethanol to remove residual NaCl on the surface. PDMS sponges were also fabricated without the addition of PDMS-*b*-PEO using the same steps. (B) Porosity comparison of PDMS sponges reveals no significant effect of incorporating surfactant. Average porosity values (%) for each sponge type are shown on top of corresponding PDMS-PEO bars. Data represents the mean ± standard deviation (*n* = *3*). (C) Hydrophilic property of the 82% porous sponge depicting the absorption of a 10 μL droplet of DI water. (D) A reduction in compressive modulus is observed as porosity increases due to an increased volume of void space within the sponge. Data represents the mean ± standard deviation (*n* ≥ 4). (E) Respective SEM images of PDMS sponges with and without PDMS-*b*-PEO (PEO). Scale bar magnifications represent 500 μm for all images.

The addition of PEO increases the hydrophilicity of the PDMS sponge, which is particularly beneficial for applications involving disinfection of needleless connectors. Increased hydrophilicity improves fluid uptake and spreading within the porous network, allowing for more efficient delivery of NO to the connector surface. This ensures better contact with aqueous contaminants and biofilms, which often reside in hydrated microenvironments. Moreover, a more hydrophilic surface enhances the interaction between the sponge and the typically hydrophilic polymer surfaces of needleless connectors, potentially improving mechanical conformability and antimicrobial efficacy. Notably, many commercial medical-grade foams, such as those used in wound dressings or cleansing swabs, are intentionally made hydrophilic to improve fluid interaction, lending further support to this material choice.

### 3.2 Polydimethylsiloxane Sponge Characterization

Porosity is a crucial factor in shaping the properties of sponge materials, influencing their suitability for specific applications. High-porosity sponges have proven valuable for facilitating cell adhesion and enhancing their capabilities to load antimicrobial agents and absorb liquids effectively.^32,33^ On the other hand, elevated porosities can hinder the formation of conductive pathways and compromise the mechanical integrity of the material, primarily due to the increased void space within the sponge.^31^ Increasing the amount of NaCl during fabrication (from 16 to 48 g) resulted in a sponge with heightened porosity, ranging from ∼60 to 82%, with the addition of the surfactant having limited impact on porosity (Figure 1B). Porosity values were also obtained using 70% IPA to reflect the solvent environment used in SNAP loading and in vitro studies. While overall comparable to water-based trends, the IPA-derived porosities were slightly lower, likely due to the solvent’s lower polarity and surface tension, which may reduce infiltration into smaller pores within the PDMS matrix (Figure S1). The porosity values derived from the water-based study were used to address the various sponge types throughout duration of the study. A UATR-FTIR analysis was performed on both PDMS and PDMS-PEO sponges to validate the successful addition of the surfactant to the material. The subtraction spectra exhibited minimal differences, except in the fingerprint region. This can be attributed to the structural similarities between PDMS and hydroxy-terminated PDMS, as well as the low concentration of the surfactant used. However, similar peaks observed in the subtraction spectra can be attributed to the incorporation of PDMS-*b*-PEO (Figure S2).^25^

In addition, the use of PDMS-*b*-PEO in PDMS films has previously been shown to enhance and maintain hydrophilicity over extended periods.^34^ This hydrophilic effect was further supported by static water contact angle measurements (Figure S3). The control PDMS sponge (82% porous) exhibited a contact angle of 118 ± 7.02°, consistent with the inherently hydrophobic nature of PDMS and in agreement with previously reported values for porous PDMS materials (Figure 1C).^35^ In contrast, the 82% porous sponge containing PEO rapidly absorbed water droplets across multiple surface locations (Video S1), confirming the successful incorporation of the surfactant and the resulting increase in surface hydrophilicity. The slight water absorption observed in the control sponge after 14 min can be attributed to the high porosity of the material.

The compressive modulus of the hydrophilic sponges is governed by porosity and characterizes the material’s stiffness and resistance to deformation under compressive load. Uniaxial compression testing was conducted to evaluate the mechanical properties of the various sponge formulations. The sponges underwent a 50% strain at a rate of 1.3 mm min^–1^, and stress–strain relationships were analyzed. The 60% porous sponge exhibited a compressive modulus of ∼105 MPa, indicating its ability to withstand compressive forces (Figure 1D). As expected, an increase in porosity resulted in a reduction in the material stiffness and compressive modulus due to a larger pore volume within the material.^31^ The 82% porous sponge displayed a significantly lower modulus of ∼19.8 MPa compared to the 60% porous sponge (*p* < 0.0001), indicating a decrease in material stiffness.

The observed trend in sponge porosity aligns with previous literature, indicating that an increase in porogen concentration results in a sponge with higher porosity.^25,36^ Although increased concentrations of PDMS-*b*-PEO in the sponge formulation can effect porosity,^26^ the limited amount of surfactant used ensures that sponge porosity remains mostly unaffected. These findings, along with the relevant peaks identified in the UATR-FTIR analysis, demonstrate that PDMS sponges can be made hydrophilic without significantly obstructing their porosity. In addition, the compressive modulus for the sponges highlights the tunability of their mechanical properties. This trend has been observed in previous literature, which reported similar compressive modulus values for PDMS sponges with comparable porosities, resulting from sugar particles as the porogen.^37^ This demonstrates that the choice of porogen used in fabricating PDMS sponges does not significantly impact their compressive strength.

Understanding the porosity and macroscopic network structures are key factors influencing the sponge’s ability to absorb and facilitate gas exchange. To investigate the cross-sectional morphology of the sponges, SEM imaging was employed, revealing the highly porous nature of the sponges (Figure 1E). The characterization of pores entailed examining pore size distributions at 30 different sites for each sponge formulation. Despite the occurrence of various interactions during fabrication, the addition of surfactant to the sponge did not yield a significant difference in the average pore size (Table S1). Therefore, it can be inferred that the alteration in porosity is not influenced by the size, but rather by the quantity of pores and interconnected tunnels. Previous literature has reported similar ranges of pore sizes with PDMS sponges formed via NaCl template removal techniques.^31,38^

### 3.3 Characterization of 70% Isopropanol and *S*-Nitroso-*N*-acetylpenicillamine Sponges

#### 3.3.1 Absorption Capacity of 70% Isopropanol Impregnated Sponge

Accurately measuring the loading capacity of 70% IPA within the sponge is essential for establishing a precise relationship between the amount of IPA added and the antimicrobial effectiveness of the sponge. Moreover, this study offers valuable insights into how sponge porosity impacts absorption capacity. This study subjected hydrophilic sponge samples to a 70% IPA solution to examine their capacity to expand within a porous PDMS substrate. The introduction of SNAP to the 70% IPA solution resulted in a green pigmentation in the sponge, signifying the successful integration of SNAP into the material. Both the 70% IPA and SNAP-IPA treated sponges displayed an increase in size (Figure 2A). The absorption capacity of each sponge was assessed by measuring the change in weight of the sponge following various soaking durations. All sponge types reached a maximum absorption capacity after 15 min in 70% IPA solution containing SNAP (Figure 2B).

**Figure 2:**
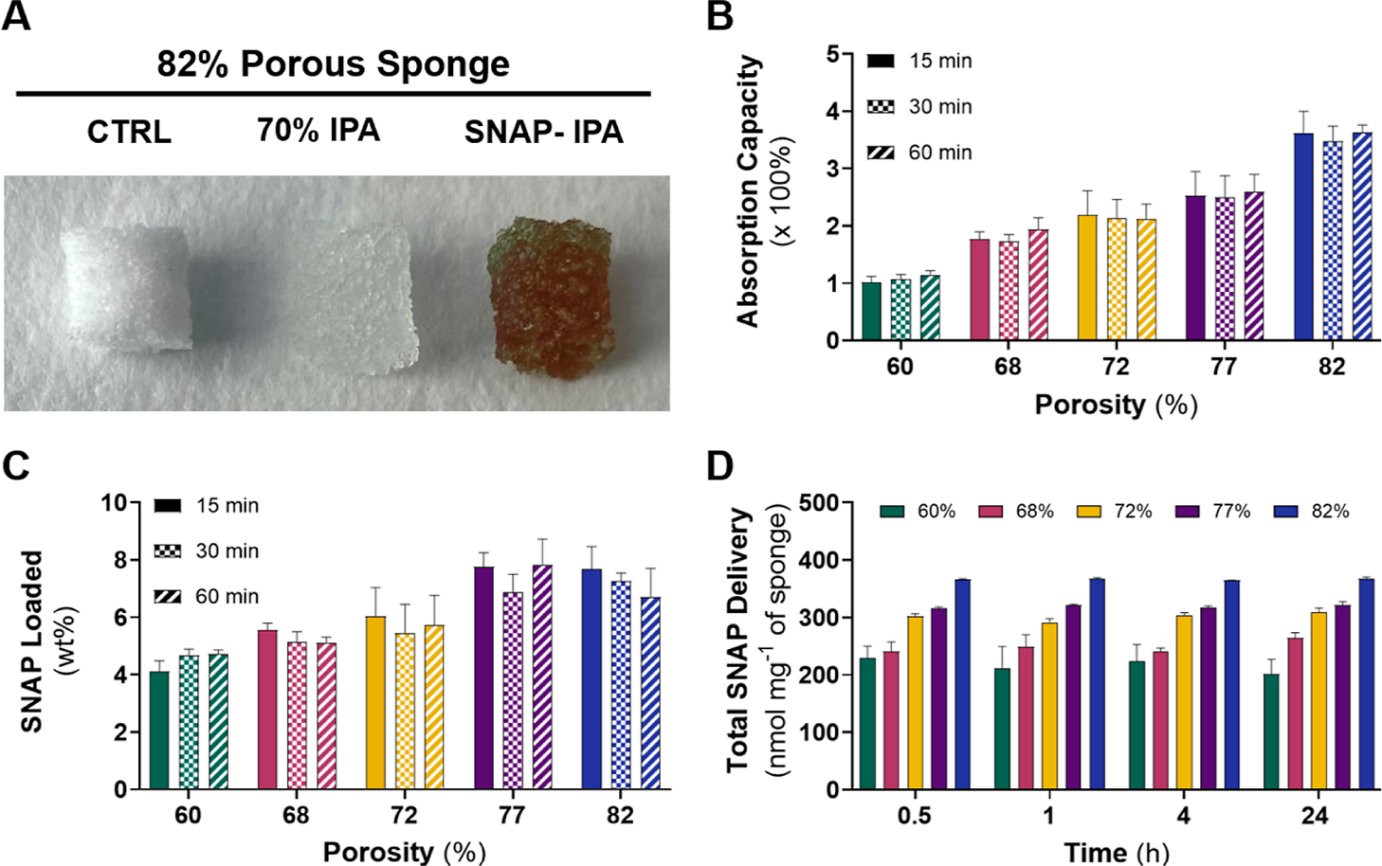
Absorption capacity and NO characteristics of the hydrophilic sponges. (A) Representative image of 82% porous sponge samples (control, IPA swollen, and SNAP-IPA swollen). Higher porosity increases the (B) absorption capacity of 70% IPA and the (C) SNAP loading capacity of hydrophilic sponges and shows no significant increase after 15 min (patterns: solids represent 15 min, checkers represent 30 min; stripes represent 60 min). (D) Secure connection of the luer cap-containing sponge releases SNAP and IPA as it is compressed by the luer connector. The assessment of SNAP released in a catheter model offers vital insights into SNAP stability within a clinical environment. Data represents the mean ± standard deviation (*n* = 3).

The absorption capacity of the sponges ranged from ∼99 to 356% for the 60 and 82% porous sponges after 15 min, respectively, demonstrating the influence of porosity on absorption behavior, with no significant difference observed upon the addition of SNAP (Figure S4). Varying the porosity of sponges significantly impacts the material’s absorption ability and, indirectly, its NO donor properties. Increased volumes of 70% IPA into the sponge allow for heightened NO release, providing higher concentrations of antimicrobial agents for more effective decontamination of needleless connectors.

Despite the well-established use of 70% IPA as a disinfecting agent, there is a noticeable absence of relevant data in the literature concerning its absorption characteristics in various polymers, specifically with PDMS. Incorporating various components into PDMS sponges may lead to slight variations in their absorption capacities. Previous research demonstrated that a PDMS/carbonized bacterial cellulose sponge exhibited an isopropanol absorption capacity of 392%, which closely aligns with the absorption capacity of the 82% porous sponge with 70% IPA.^39^ While the absorption capacity of PDMS sponges using IPA as the solvent, especially 70% IPA, has not been extensively studied, absorption capacities in the range of hydrophilic PDMS sponges using other alcohols, such as methanol and ethanol, have been examined, with capacities reaching around 300%.^31,40^ Commercially available sponges are typically crafted from polyether urethane materials owing to their excellent absorption capabilities. Nevertheless, PDMS offers superior gas permeability, particularly for NO, in comparison to polyurethanes, enabling more efficient diffusion of NO throughout a PDMS sponge.^41^

#### 3.3.2 Integration of *S*-Nitroso-*N*-acetylpenicillamine into Polydimethylsiloxane Sponge

The SNAP-incorporated sponge was designed to serve as a highly efficient disinfecting agent within luer caps through the dual diffusive action of NO and 70% IPA. To understand the release of NO from the sponge and its resulting antimicrobial activity, the SNAP loading capacity within the sponge was determined. To optimize SNAP loading in the sponges, the solubility of SNAP in 70% IPA was investigated. A concentration of 85 mg mL^–1^ of SNAP in 70% IPA was employed for further examination, as higher concentrations led to rapid evaporation and precipitation. Quantification of SNAP in each sponge type was determined by weighing the sample post-absorption and then extracting all of the loaded SNAP into fresh 70% IPA before analysis using UV–vis. The sponges reached a maximum SNAP loading ability (Figure 2C) after 15 min in a SNAP-IPA solution. Similarly, as porosity increased, the SNAP loading capacity of sponges also increased, ranging from ∼4.12 to 7.68 wt % for the 60 and 82% porous sponges after 15 min. No significant increase in SNAP loading was observed beyond 15 min for all sponge types.

Despite a steady increase in IPA absorption across sponges with increasing porosity, SNAP loading did not rise proportionally and appeared to plateau at higher porosity levels. Specifically, while SNAP loading increased in sponges with low to moderate porosity, it remained similar for the two highest porosity sponges, despite greater IPA uptake. This behavior suggests that SNAP uptake is not governed solely by solvent absorption capacity, but also by factors such as limited diffusion into the PDMS matrix or saturation of available interaction sites within the polymer network. Nonetheless, the 82% porous sponge was selected for continued use due to its significantly enhanced fluid uptake, pore interconnectivity, and surface contact potential, all advantageous features for maximizing NO delivery and antimicrobial coverage in the intended disinfection application.

Silicone sponges have been used for drug loading and delivery purposes. However, the incorporation of an RSNO in a sponge has limited exploration. In a recent study, a wound-healing cold-pressed collagen sponge was loaded with *S*-nitrosoglutathione (GSNO) to release NO as it absorbed exudate from the surrounding wound.^24^ However, due to variability in exudate diffusion, humidity, and other nonstandardized factors, a challenge arises when measuring NO release in such a system, making the quantity of SNAP loaded into these PDMS sponges a novel addition to the literature. While direct comparison of SNAP loading ability, or any NO donor loading ability, of PDMS sponges with existing research is limited, it is worth noting that SNAP has been successfully incorporated into a variety of polymers and medical device prototypes with enhanced stability and long-term NO-release.^42^ Previous investigations have shown that generating porous structures in polyurethane films results in enhanced SNAP loading compared to solid samples devoid of pores.^43^ This study yielded similar findings, that introducing porosity enhances the PDMS’s capacity to incorporate more SNAP compared to other solid silicone substrates lacking pores. For instance, a silicone polymer tubing achieved ∼5 wt % of SNAP loading with a 125 mg mL^–1^ concentration of SNAP in tetrahydrofuran (THF).^44^ The 77 and 82% porous sponges could load ∼7.77 and 7.68 wt % of SNAP (*p* > 0.05), exhibiting ∼53% more SNAP loaded into the polymer matrix compared to the silicone tubing.

The SNAP loading ability of hydrophilic PDMS sponges demonstrates that increasing sponge porosity facilitates greater incorporation of SNAP into the polymer material. Notably, there is a saturation point in SNAP loading after 15 min. For example, the 60% porous sponge exhibited a ∼0.61 wt % rise in SNAP loading from 15 to 60 min (*p* > 0.05), while the 82% porous sponge actually showed a slight reduction in SNAP loading by ∼0.97 wt % (*p* > 0.05). For this reason, subsequent experiments followed a 15 min absorption period. This trend among sponge types signifies that the maximum incorporation of SNAP into the sponge pores and PDMS matrix happens quickly and becomes saturated within 15 min of exposure.

#### 3.3.3 *S*-Nitroso-*N*-acetylpenicillamine Delivery from Polydimethylsiloxane Sponge

The delivery of SNAP from each sponge was evaluated within a simulated catheter setup. This model was designed to replicate real-world conditions commonly encountered in healthcare settings, particularly when using catheters equipped with needleless connectors. This study aimed to understand the release of SNAP from sponges with varying porosities by evaluating the amount of SNAP remaining in the sponge over a 24 h period. While disinfecting caps have shown effectiveness over multiple days,^12,45^ the ability to disinfect the hub regions of catheters quickly and effectively was specifically investigated, considering that these connectors are frequently accessed multiple times a day in healthcare settings.

Sponges infused with the SNAP-IPA solution for 15 min were immediately placed into luer caps and secured onto luer connectors attached to a catheter model. The quantity of SNAP delivered to the luer connectors was tested under real-world conditions by monitoring the amount of SNAP remaining in the sponge after 0.5, 1, 4, and 24 h following methods taken in Section 2.9 to quantify SNAP loading. All sponge formulations exhibited a similar trend in SNAP delivery (Figure 2D) over the 24 h period. The results depict a strong correlation between sponge porosity and SNAP delivery.

The 82% porous sponge was able to release ∼368 nmol mg^–1^ of SNAP within 24 h, releasing significantly higher levels compared to the other sponge types (*p* < 0.05). These values were calculated using the measured amount of SNAP loaded into each sponge and the quantity of SNAP remaining in each sponge through extraction in 10 mL 70% IPA. The high levels of SNAP delivered to the luer connector were observed by the lack of green pigmentation in the sponges after the designated period of time. The amount of SNAP released did not increase significantly over time in any of the sponge types. This can be attributed to the compression of the sponges when attached to luer connectors, which causes a fast release of SNAP and IPA. The 82% porous sponge exhibited the highest SNAP release among the sponge formulations due to its higher porosity and heightened SNAP concentration.

The release of SNAP from all sponge types showed a high initial release rate with no significant increase after 30 min. This initial burst is observed due to the sponge being compressed in order to release the antimicrobial agents. The amount of SNAP remaining in the sponge was used to determine the quantity of SNAP released into the luer connector with the 60% porous sponge releasing ∼229 nmol mg^–1^ and the 82% porous sponge releasing ∼366 nmol mg^–1^, respectively, within the first 30 min. Due to the specific application of this technology, literature lacks leaching of NO donors via squeezing of the material. However, porous films loaded with ∼19.6 and 14.0 wt % SNAP released ∼18 and 35% of SNAP when subjected to a PBS solution at 37 °C.^43^ Although PDMS sponges were subjected to a different environment, the 60 and 82% porous sponges released significantly higher quantities of SNAP, releasing ∼94.94 and 97.25% of their total SNAP loaded.

While a previously reported NO-releasing sponge demonstrated NO release kinetics using GSNO as the NO donor and an NOA,^24^ NO release from SNAP-IPA sponges examined in the catheter model was used as an indirect method of quantifying NO released over time. However, to ensure NO was released from the sponges, instantaneous NO release from the 82% porous sponge, following Supporting Information Methods S1.1, was examined using an NOA at 37 °C. SNAP-IPA samples were quickly wrapped in Tegaderm to prevent IPA from leaching while allowing NO to escape and be accurately quantified during the study. The application of this technology lies in the sponge’s ability to release IPA and NO when compressed, which cannot be done within an NOA as the process is conducted in a closed system. However, the limited studies using an NOA confirmed the release of NO from the 82% porous sponge (Figure S5). Overall, these findings highlight the distinctive release of SNAP exhibited by PDMS sponges of varying porosities.

The sponge serves not only as a reservoir for SNAP-IPA, but also as a mechanically active and spatially targeted delivery device for disinfection of needleless connectors. Unlike catheter lock solutions, which are confined to disinfecting the inner lumen of the catheter, the sponge-based system is designed to address external contamination at the luer connector surface. The compressive properties of the sponge allow it to conform to and remain in contact with the connector, enabling consistent, localized delivery of NO and IPA without requiring active handling or liquid instillation. This simplifies the workflow in clinical settings by reducing labor, eliminating the need for aspiration, and allowing for passive disinfection during usage or between access. Additionally, the sponge format allows for preloading of antimicrobial agents, offering logistical advantages over solutions that require on-demand preparation or careful handling. This added functionality and usability make the sponge a more practical and clinically relevant platform for surface disinfection in catheter care.

#### 3.3.4 Stability of *S*-Nitroso-*N*-acetylpenicillamine-Isopropanol Sponge

The storage stability of SNAP-IPA sponges is critical to ensuring their practical use in healthcare settings. To evaluate this, 82% porous SNAP-IPA sponges were stored in sealed luer caps (wrapped in foil and parafilm) at room temperature, 4 °C, or −20 °C in the dark. After 1, 4, or 7 days, sponges were dried in a vacuum desiccator overnight (protected from light) and leached in PBS with EDTA at 37 °C for 24 h. The resulting leachates were analyzed to quantify residual SNAP, as SNAP is known to degrade over time. Sponges stored at −20 °C retained the highest SNAP levels, consistent with prior studies showing enhanced SNAP stability at subzero temperatures (Figure S6A).^46^ In contrast, reduced SNAP content was observed in sponges stored at 4 °C and room temperature, indicating degradation over time under those conditions.

Nitric oxide release was assessed using the same leachates via the Griess assay, which measures nitrite accumulation as an indirect indicator of NO release. Higher nitrite levels were detected in sponges stored at −20 °C, corresponding with greater SNAP availability (Figure S6B). After 7 days, sponges stored at −20 °C produced 0.39 μg nitrite/mg sponge (∼0.69 mM), whereas those stored at 4 °C and room temperature released 0.35 and 0.26 μg nitrite/mg sponge, respectively. Although the Griess assay does not provide a direct quantification of NO release, due to variation in how materials convert NO to nitrite, it remains as a useful analysis method. Similar nitrite concentrations in the mM range have been reported for other SNAP-releasing materials.^47^ The observed trends in both SNAP stability and nitrite production support enhanced preservation of SNAP at lower storage temperatures.

Additionally, sponge mass was measured before and after storage. A slight, nonsignificant decrease in sponge mass was observed for samples stored at 4 °C and room temperature, potentially due to SNAP degradation (Figure S6C). All samples showed a modest weight loss (7–9%) overall, which can primarily be attributed to rapid IPA evaporation during weighing. However, the consistency of this small loss across conditions suggests that IPA was not actively evaporating during storage.

### 3.4 Antimicrobial Efficacy via Zone of Inhibition Assay

Antimicrobial agents like 70% IPA are renowned for their efficacy in combating bacterial infections.^12^ Given the rapid formation and colonization of biofilms, particularly on surfaces like catheters,^48,49^ the introduction of NO as an additional diffusive agent can complement the action of 70% IPA, potentially strengthening efforts to prevent and eradicate biofilms. In this initial investigation, a standard zone of inhibition (ZOI) test was conducted to examine the antimicrobial effects of SNAP-IPA and 70% IPA alone compared to pristine samples with no antimicrobial activity. Hydrophilic sponges were infused with either 70% IPA or SNAP-IPA for 15 min before being placed onto microbial agar plates, with subsequent comparison to control sponges without either antimicrobial agent incorporated. To achieve maximum antimicrobial potential, the 60% porous sponge was excluded from the antimicrobial studies, as its low porosity (Figure 1B) and corresponding SNAP loading (Figure 2C) and release (Figure 2D) were expected to result in limited antimicrobial activity. The quantitative results demonstrated consistent trends across sponge types, microbes, and antimicrobial agents (Table S2).

When immersed in the combined SNAP-IPA solution, hydrophilic PDMS sponges exhibited a significantly heightened microbiocidal activity against all the tested microbes compared to 70% IPA alone. The antibacterial efficacy displayed noteworthy variations between the two experimental groups, with 100% of sponges showcasing substantial disparities against a broad spectrum of bacteria, including Gram-negative *E. coli* and *P. aeruginosa*, as well as Gram-positive *S. aureus* and *S. epidermidis* (Figure 3A–D). The variability in antibacterial performance can be attributed to their distinct membrane properties.^50^ The SNAP-IPA sponges exhibited a progressively improved microbial inhibition effect as the sponge’s porosity increased. However, this advancement in antimicrobial impact was not observed for the IPA sponges, implying that the microbiocidal capacity of 70% IPA might be restricted (even though the absorption capacity increased, Figure 2B).

**Figure 3:**
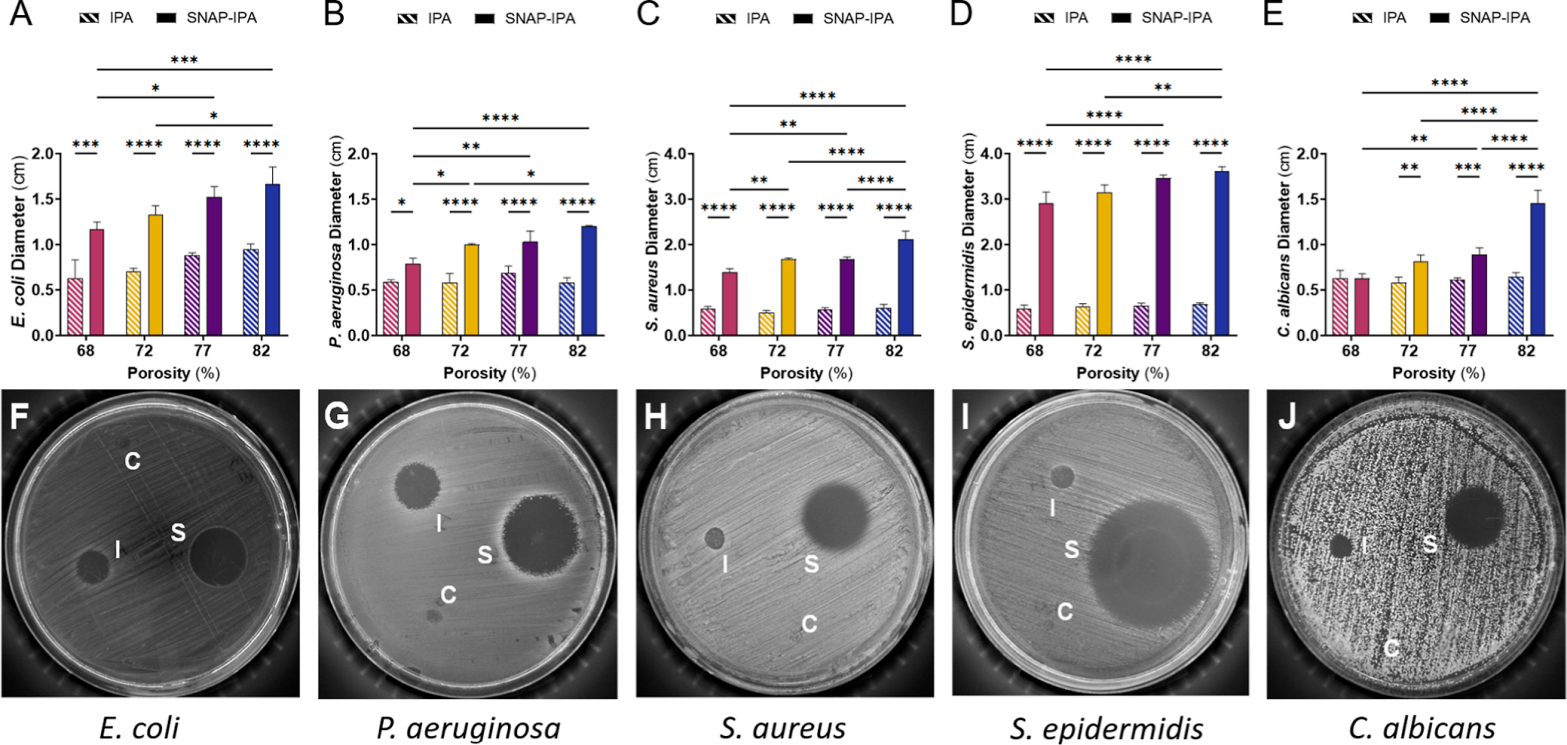
Zone of inhibition comparison between 70% IPA and SNAP-IPA swollen sponges. Quantitative data showcasing inhibited zones of each microbe: (A) *E. coli*, (B) *P. aeruginosa*, (C) *S. aureus*, (D) *S. epidermidis*, (E) *C. albicans*. Striped bars represent 70% IPA swollen sponges and solid bars represent SNAP-IPA swollen sponges. Complementing representative images of each microbe tested: (F) *E. coli*, (G) *P. aeruginosa*, (H) *S. aureus*, (I) *S. epidermidis*, (J) *C. albicans*, demonstrating significant differences upon SNAP addition. Image labels indicate where control sponges (C), 70% IPA swollen sponges (I), and SNAP-IPA swollen sponges (S) were placed. Disrupted microbial growth areas represent where control sponges were placed with unquantifiable zones. Data represents the mean ± standard deviation (*n* ≥ 3). *Indicates significance: * (*p* < 0.05), ** (*p* < 0.01), *** (*p* < 0.001), **** (*p* < 0.0001).

Bacterial growth inhibition using the 82% porous sponge resulted in inhibition diameters of ∼2.12 and 3.61 cm against *S. aureus* and *S. epidermidis*, respectively, while for *E. coli* and *P. aeruginosa*, diameters measured ∼1.66 and 1.20 cm, respectively (Figure 3F–I). The larger inhibition zones observed against Gram-positive bacteria (*S. aureus* and *S. epidermidis*) compared to Gram-negative bacteria (*E. coli* and *P. aeruginosa*) were anticipated, given that the outer membrane of Gram-negative bacteria makes it more challenging for NO to permeate the cell.^51^ The antibacterial attributes of NO stem from its reactive-oxygen species derivatives, including but not limited to nitrogen dioxides and peroxynitrites, which induce nitrosative and oxidative stress on various microbial components.^19^ Nonetheless, these potent mechanisms of microbial eradication are not always sufficient against some microbes, such as *C. albicans*, which possess an inducible NO defense mechanism.^52^

Prior research has indicated that NO alone may lack antifungal properties,^51^ potentially due to variations in the concentration of the NO donor employed. While the observed antifungal activity might stem from a synergistic interplay between NO and 70% IPA, it may be that the enhanced antifungal effect is primarily attributed to the elevated concentration of SNAP within the sponges. Several factors support this perspective. Despite 70% IPA displaying antifungal activity against *Candida* species,^53,54^ the lack of significant differences among the various sponge types suggests that an increase in 70% IPA loading is not the primary driver behind this fungal inhibition (Figure 3E). Notably, the significant disparities observed within the SNAP-IPA sponges, specifically the 82% porous sponge’s greater fungal inhibition compared to the other sponge types (Figure 3J), strongly suggests that the concentration of NO within the sponges governs their antifungal activity. Moreover, the 68% porous sponge, containing the lowest SNAP content, fails to demonstrate enhanced antifungal activity compared to 70% IPA alone, providing further evidence of the importance of NO concentration in dictating the sponge’s antifungal potential.

This consistent pattern of antimicrobial activity observed with 70% IPA swollen sponges against *C. albicans* holds across all the tested microbes. Remarkably, there is no discernible enhancement in microbial inhibition against any of the tested microbes as the sponge’s porosity and 70% IPA absorption capacity increase. Given IPA’s rapid evaporation upon exposure to air, prolonged release of the disinfectant could potentially yield more effective results in this particular application. However, the application of 70% IPA has been examined in a similar ZOI study, showing that the inhibited zone diameters closely resemble those observed in sponges saturated in 70% IPA, specifically concerning Gram-positive bacteria and fungi.^13^ These findings provide crucial insight into the antimicrobial efficacy of NO and its donor molecule, SNAP, particularly considering that the antifungal potential of NO has shown limited ability to hinder fungal growth significantly. Further investigations delving into the precise interaction and collaborative mechanism of SNAP and 70% IPA may unveil intriguing insights in modulating the antifungal properties of SNAP, thereby enhancing its viability for diverse biomedical applications.

### 3.5 Antimicrobial Efficacy via Contact Killing Study

Planktonic microorganisms that come into contact with medical devices have the potential to establish biofilms rapidly.^55^ Recognizing the critical importance of the initial hours in the biofilm formation process, a 4 h exposure study was conducted against *E. coli*, *S. aureus*, and *C. albicans*, as representative Gram-negative bacteria, Gram-positive bacteria, and fungi, respectively. These microorganisms are linked to a wide range of device-related infections with *E. coli* associated with urinary tract infections, *S. aureus* with implant-related infections, and *C. albicans* with left ventricular assist device (LVAD) and catheter-related infections.^56,57^ The 82% porous sponge was selected for this study due to its significant antimicrobial activity observed in the ZOI study. After subjecting sponge samples (control, 70% IPA swollen, SNAP-IPA swollen) to a microbial suspension for 4 h, the reduction in viable CFUs was determined and normalized to an average swollen sponge volume (Figure 4A–C). Normalizing by volume or weight, rather than surface area, is particularly appropriate for porous materials like sponges, where surface area is difficult to define and can vary more significantly; this approach has also been supported in prior literature.^24,58^ In both *E. coli* and *C. albicans*, 70% IPA had no significant effect in killing the microbes in solution and only exhibited a slight reduction in viable *S. aureus*. The improved microbicidal activity of the NO-releasing sponge provided increased killing against all microbes compared to control and 70% IPA control sponges exhibiting a ∼0.53, 3.00, and 0.67-log reduction in viable *E. coli*, *S. aureus*, and *C. albicans*, respectively (Table S3). As discussed previously, the increased killing ability observed with *S. aureus* compared to the other microbes can be attributed to the difference in membranous properties and specific defense mechanisms each microbe withholds.

**Figure 4:**
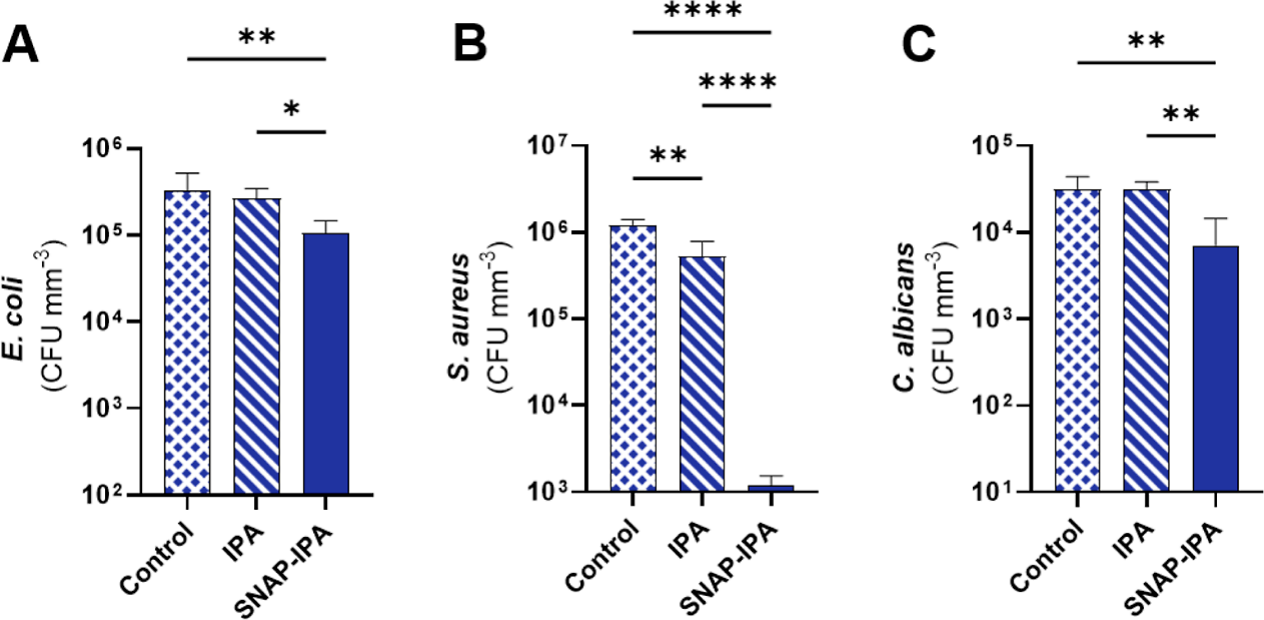
Microbial viability after 4 h exposure to 82% porous PDMS sponges (control, 70% IPA swollen, and SNAP-IPA swollen). Viable planktonic microbes presented as CFU per mm^3^ of sponge: (A) *E. coli*, (B) *S. aureus*, (C) *C. albicans*. Data represents the mean ± standard deviation (*n* = 4). * Indicates significance: * (*p* < 0.05), ** (*p* < 0.01), **** (*p* < 0.0001).

Biofilm formation involves several critical stages, including microbial dispersion in its planktonic state, adhesion to surfaces, and proliferation, resulting in the development of a protective biofilm encased in an extracellular polymeric matrix that shields the cells from antibiotic and disinfectant treatments.^55^

Since microbial adhesion and biofilm formation mark the final stages leading to infections associated with medical devices, existing literature primarily focuses on the antiadherence and biofilm eradication capabilities of NO in combination with other antimicrobial agents.^59–61^ Surprisingly, the antimicrobial efficacy of NO against planktonic microorganisms has not received extensive attention in research.

The results of this study align with previously reported literature in which NO’s antibacterial activity against planktonic forms of *E. coli* and *S. aureus* was examined using a 125 mg mL^–1^ concentration of SNAP. Following a 24 h exposure to bacteria, SNAP films demonstrated ∼1.20 and 2.26 log reductions in viable *E. coli* and *S. aureus* *.* ^62^ A more pronounced antibacterial effect on *S. aureus* compared to *E. coli* was seen in both studies, further highlighting Gram-positive bacteria’s heightened susceptibility to NO compared to Gram-negative bacteria. Previous literature highlights the synergistic antimicrobial effects achieved by NO-releasing technology with other antimicrobial agents.^51^ However, combining SNAP with the antifungal agent amphotericin B does not significantly enhance antifungal activity compared to amphotericin B alone.^63^ In contrast, the combination of SNAP with 70% IPA demonstrated a substantial improvement in antifungal activity compared to 70% IPA alone (*p* < 0.01).

The inclusion of 70% IPA may impact the antibacterial activity of NO in solution. However, as 70% IPA is commonly employed as a disinfectant for removing adhered microbes, relevant data on the antimicrobial agent’s ability to kill microbes in solution is scarce. The lack of significant microbicidal activity associated with 70% IPA might be attributed to its further dilution in solution, causing it to lose its microbicidal properties. Nonetheless, observations suggest that 70% IPA does not significantly hinder microbial growth or promote the killing of microbes in solution, leaving NO as the primary driver of substantial antimicrobial activity in the sponge.

### 3.6 In Situ Microbial Disinfection Study

Microbes can proliferate and disseminate within the inner lumen of catheters, stemming from sources of contamination such as the skin around catheter insertion sites and needleless connectors. These connectors, widely used for vascular access, are prone to contamination from frequent handling without consistent disinfection.^64^ The passive disinfection method of protective caps has demonstrated a significant reduction in CRBSIs compared to active disinfection techniques like wiping.^65^ *E. coli*, *P. aeruginosa*, *S. aureus*, *S. epidermidis*, and *C. albicans* were tested due to their common association with biofilm formation and medical device infections.^66^ Given the superior antimicrobial performance of SNAP and 70% IPA over 70% IPA alone, the SNAP-IPA swollen sponge was expected to effectively disinfect precontaminated needleless connectors. Microbes were allowed to adhere to connectors for 6 h before applying 82% porous sponges swollen with either IPA or SNAP-IPA inside luer caps. Upon securing the cap onto the contaminated connectors, the sponge was compressed, controlling the release of antimicrobial agents. To ensure consistency, sponge dimensions matched those used in SNAP delivery studies. As there was no notable increase in SNAP delivery observed beyond the initial 30 min for the 82% porous sponge (Figure 2D), disinfection was assessed for 30 min by evaluating microbial viability (Figure 5A–E).

**Figure 5:**
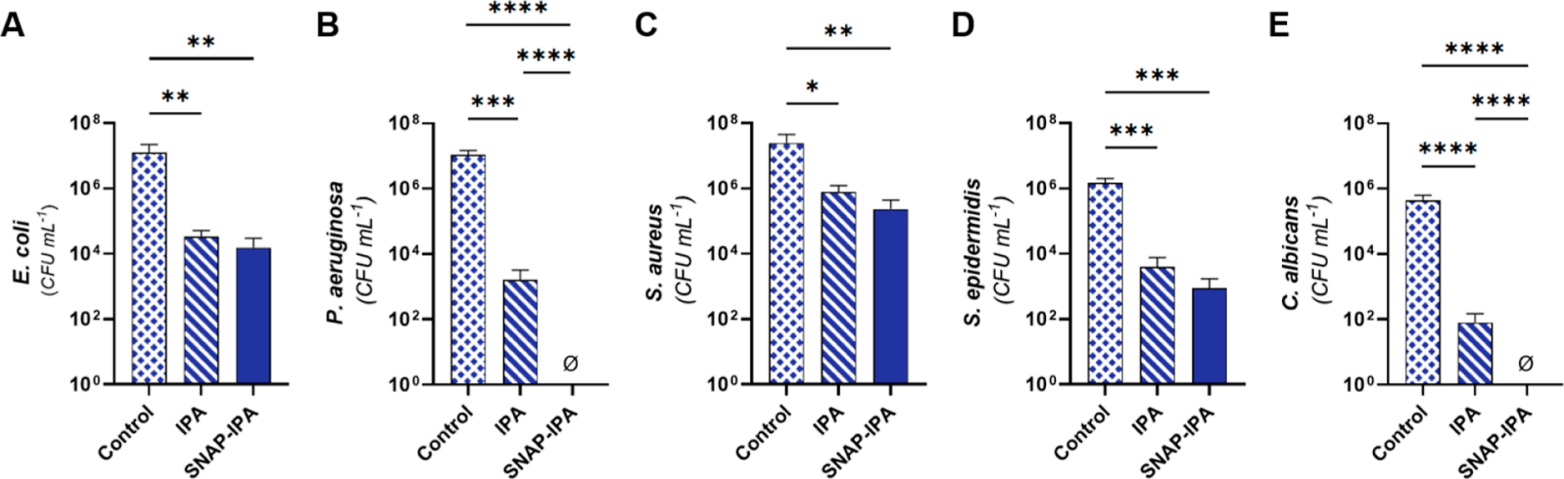
Microbial viability of contaminated luer connectors after 30 min exposure to IPA and SNAP-IPA 82% porous sponges. Viable adhered microbes presented as CFU per mL: (A) *E. coli*, (B) *P. aeruginosa*, (C) *S. aureus*, (D) *S. epidermidis*, (E) *C. albicans*. Data represents the mean ± standard deviation (*n* ≥ 4). * Indicates significance: * (*p* < 0.05), ** (*p* < 0.01), *** (*p* < 0.001), **** (*p* < 0.0001). Ø indicates results were below the detection limit.

Sponges swollen with SNAP-IPA demonstrated remarkable decontamination, achieving ∼2.91-, 7.04-, 2.02-, 3.21-, and 5.65-log reductions in viable *E. coli*, *P. aeruginosa*, *S. aureus*, *S. epidermidis*, and *C. albicans* (Table S4). The antimicrobial study focused on the effectiveness of direct contact killing via SNAP-IPA compared to IPA alone, while the planktonic study examined performance in microbial suspension. By eliminating external interactions from PBS, the disinfecting sponge effectively eradicated adhered microbes through IPA release upon compression and sustained NO release. Although NOA analysis indicated low NO release in the first hour (Figure S5), the observed antimicrobial effects confirm NO activity, as microbial viability was reduced compared to IPA sponges. While SNAP-IPA sponges did not significantly outperform IPA sponges in overall microbial viability reduction, they exhibited superior disinfection against *P. aeruginosa* and *C. albicans*.

The strong antifungal efficacy of the SNAP-IPA combination suggests a complementary effect. As a disinfectant, 70% IPA precipitates cell wall surface proteins, with the specific dilution enhancing penetration and slowing evaporation, thereby increasing antimicrobial effectiveness.^16^ Inside the cell, IPA denatures structural and enzymatic proteins, leading to cell death.^13^ By disrupting microbial cell walls, IPA may facilitate NO entry, amplifying antimicrobial effects, particularly against *C. albicans* and *P. aeruginosa*.

Increased susceptibility of *P. aeruginosa* and *C. albicans* to the NO and IPA combination may be influenced by their distinct biofilm-forming abilities and adhesion characteristics. *S. aureus*, *S. epidermidis*, and *E. coli* are known for robust biofilm formation on medical devices, which can enhance their resistance to antimicrobial treatments. In contrast, while *P. aeruginosa* and *C. albicans* can form biofilms, their structures may be more vulnerable to NO and IPA. Adhesion to medical devices can vary among these microbes. *S. aureus* and *S. epidermidis* possess surface proteins that can facilitate strong adhesion, contributing to persistent infections.^67^ *E. coli*, *P. aeruginosa*, and *C. albicans* exhibit similar capabilities, though different medical devices and materials can vary the adhesion properties of the microbe.^68^ These structural and adhesion differences may contribute to the enhanced susceptibility of *P. aeruginosa* and *C. albicans* to SNAP-IPA treatment.

Although the initial contact is critical for demonstrating antimicrobial potential and preventing early infection, it is also important that the treatment maintains its efficacy over time. To assess this, a 24 h disinfection study was conducted against *S. aureus* as a preliminary step to evaluate the potential for microbial regrowth. Following the same methods as described in Section 2.15, infected needleless connectors were capped with either IPA or SNAP-IPA sponges for 24 h. Both treatments effectively eliminated all detectable adhered bacteria, indicating that neither allowed bacterial regrowth during the incubation period (Figure S7). These findings confirm that the SNAP-IPA sponge performs as well as the established 70% IPA treatment in long-term disinfection. Importantly, SNAP-IPA had already demonstrated superior efficacy during the critical initial 30 min contact period, suggesting its added benefit in early antimicrobial action. Future studies will further investigate the durability of these effects over extended durations (e.g., up to 7 days).

Alcohol-based disinfectants have proven to be ineffective against spore-forming bacteria like *Bacillus* and *Clostridium* *.* ^69^ However, NO has been shown to mediate antimicrobial effects against various spore-forming bacteria.^70,71^ Further research into the SNAP-IPA combination could explore its effectiveness against these resilient strains contributing to HAIs.^72^ Additional investigations could assess alternative alcohols, varying concentrations, and other NO donors, such as GSNO, to enhance solubility and antimicrobial efficacy. Overall, the disinfection properties of NO and 70% IPA offer a promising approach to reducing CRBSIs and HAIs, warranting further exploration and optimization.

## 4 Conclusions

Inadequate disinfection of needleless connectors poses a serious threat of bloodstream infections. While alcohol-based disinfectants are effective, the concern for microbial resistance highlights the need to incorporate an NO donor to enhance antimicrobial effectiveness and minimize resistance. The combination of NO and 70% IPA demonstrated remarkable antimicrobial properties against various microorganisms compared to the common disinfectant, 70% IPA alone, and significantly disinfected needleless connectors. By tuning the porosity and wettability of PDMS, a sponge-like material was fabricated using a facile salt template removal technique, resulting in the enhancement of the material, including properties such as compressive strength, absorption capacity, SNAP loading, NO release kinetics, and antimicrobial efficacy. Distinct trends emerged as the sponge’s porosity increased from 60 to 82%. The PDMS sponge became more compressible with higher porosity, while its 70% IPA absorption and SNAP loading capabilities significantly improved. Consequently, the 82% porous sponge exhibited a substantial increase in SNAP delivery when subjected to a simulated catheter model, releasing high levels of SNAP within just 30 min. The impact of porosity on the sponge’s properties was reinforced by the combined antimicrobial effect of NO and 70% IPA against both Gram-positive and Gram-negative bacteria, as well as a fungal strain. The 82% porous sponge displayed impressive microbiostatic properties, evident in the zone of inhibition studies, and effectively eliminated microbes when exposed to a microbial solution.

The sponge’s rapid release of antimicrobial agents positions it as an ideal candidate for disinfecting needleless connectors. The 82% porous sponge exhibited potent disinfection capabilities against *E. coli*, *S. aureus*, and *C. albicans*, targeting a prevalent source of microbial contamination in healthcare settings. This innovative approach to incorporating and releasing antimicrobial agents introduces a novel material for preventing infections in hospitals and healthcare facilities. Moreover, the adjustable characteristics of the PDMS sponge render it versatile for a range of applications, such as tissue engineering and infection prevention, particularly at LVAD lead insertion sites.

## Supplementary Material





## Notes

Porosity comparison using water and 70% IPA; UATR-FTIR characterization of PEO incorporation; static water contact angle measurements; 70% IPA absorption capacity comparison with and without SNAP; representative nitric oxide release profile by NOA analysis; storage stability via SNAP degradation, nitrite accumulation, and change in mass; quantitative porosity and pore size values; quantitative zone of inhibition values; quantitative planktonic microbe log-reduction values; quantitative adhered microbe log-reduction values; extended disinfection results (PDF)
Water absorption video (MP4)

## References

[1] Clinical Infectious Diseases.1 October News Clin. Infect. Dis. 2011, 53, (7), i–ii 10.1093/cid/cir564. 23126012

[2] Rupp M. E., Karnatak R. (2018). Intravascular Catheter-Related Bloodstream Infections. Infect. Dis. Clin..

[3] Spengler R. F., Greenough W. B. (1978). 3rd. Hospital costs and mortality
attributed to nosocomial
bacteremias. J. Am. Med. Assoc..

[4] Moureau N. L., Flynn J. (2015). Disinfection of Needleless
Connector Hubs: Clinical Evidence Systematic
Review. Nurs. Res. Pract..

[5] Bouza E., Alvarado N., Alcalá L., Pérez M. J., Rincón C., Muñoz P. (2007). A randomized
and prospective study
of 3 procedures for the diagnosis of catheter-related bloodstream
infection without catheter withdrawal. Clin.
Infect. Dis..

[6] Cercenado E., Ena J., Rodriguezcreixems M., Romero I., Bouza E. (1990). A Conservative
Procedure for the Diagnosis of Catheter-Related Infections. Arch. Intern. Med..

[7] Buetti N., Timsit J.-F. (2019). Management and Prevention of Central
Venous Catheter-Related
Infections in the ICU. Semin. Respir. Crit.
Care Med..

[8] Centers for Disease Control and Prevention , How Antibiotic Resistance Happens. 2020. https://www.cdc.gov/drugresistance/about/how-resistance-happens.html (accessed July 24, 2023).

[9] Simon A., Fahrendorf W., Hitschmann G. (2021). Preclinical evaluation of passive
disinfection caps with a long-term catheter for the prevention of
catheter-related bloodstream infection in pediatric cancer patients. GMS Hyg. Infect. Contr..

[10] Mazher M. A., Kallen A., Edwards J. R., Donlan R. M. (2013). An evaluation of
disinfection protocols used for needleless connectors of central venous
catheters. Lett. Appl. Microbiol..

[11] Adams D., Quayum M., Worthington T., Lambert P., Elliott T. (2005). Evaluation
of a 2% chlorhexidine gluconate in 70% isopropyl alcohol skin disinfectant. J. Hosp. Infect..

[12] Casey A. L., Karpanen T. J., Nightingale P., Elliott T. S. J. (2018). An in vitro comparison
of standard cleaning to a continuous passive disinfection cap for
the decontamination of needle-free connectors. Antimicrob. Resist. Infect. Control.

[13] Thaddeus N. I., Francis E. C., Jane O. O., Obumneme A. C., Okechukwu E. C. (2018). Effects
of some common additives on the antimicrobial activities of alcohol-based
hand sanitizers. Asian Pac. J. Trop. Med..

[14] Newman, T. Bacteria are becoming resistant to alcohol-based disinfectants. Medical News Today. 2018 . https://www.medicalnewstoday.com/articles/322646.

[15] Hamad
Vuai S. A., Sahini M. G., Sule K. S., Ripanda A. S., Mwanga H. M. (2022). A comparative in-vitro study on antimicrobial efficacy
of on-market alcohol-based hand washing sanitizers towards combating
microbes and its application in combating Covid-19 global outbreak. Heliyon.

[16] Global, M. Why Is 70% Isopropyl Alcohol (IPA) a Better Disinfectant than 99% Isopropanol, and What Is IPA Used For?; PAC, 2017.

[17] Barraud N., Hassett D. J., Hwang S. H., Rice S. A., Kjelleberg S., Webb J. S. (2006). Involvement of nitric
oxide in biofilm dispersal of
Pseudomonas aeruginosa. J. Bacteriol..

[18] Änggård E. (1994). Nitric oxide:
mediator, murderer, and medicine. Lancet.

[19] Fang F. C. (1997). Perspectives
series: host/pathogen interactions. Mechanisms of nitric oxide-related
antimicrobial activity. J. Clin. Invest..

[20] Wo Y. Q., Li Z., Colletta A., Wu J. F., Xi C. W., Matzger A. J., Brisbois E. J., Bartlett R. H., Meyerhoff M. E. (2017). Study of
crystal formation and nitric oxide (NO) release mechanism from *S*-nitroso-*N*-acetylpenicillamine (SNAP)-doped
CarboSil polymer composites for potential antimicrobial applications. Composites, Part B.

[21] Pant J., Goudie M. J., Chaji S. M., Johnson B. W., Handa H. (2018). Nitric oxide
releasing vascular catheters for eradicating bacterial infection. J. Biomed. Mater. Res., Part B.

[22] Field L., Dilts R. V., Ravichandran R., Lenhert P. G., Carnahan G. E. (1978). An unusually
stable thionitrite from N-acetyl-D, L-penicillamine; X-ray crystal
and molecular structure of 2-(acetylamino)-2-carboxy-1, 1-dimethylethyl
thionitrite. J. Chem. Soc. Chem. Commun..

[23] Chug M. K., Brisbois E. J. (2022). Recent Developments in Multifunctional
Antimicrobial
Surfaces and Applications toward Advanced Nitric Oxide-Based Biomaterials. ACS Mater. Au.

[24] Póvoa V. C. O., Dos Santos G. J. V. P., Picheth G. F., Jara C. P., da Silva L. C. E., de
Araújo E. P., de Oliveira M. G. (2020). Wound healing
action of nitric oxide-releasing self-expandable collagen sponge. J. Tissue Eng. Regener. Med..

[25] Parameswaran C., Chaudhary R. P., Hosangadi Prutvi S., Gupta D. (2022). Rapid One Step Fabrication
of Hydrophilic Hierarchical Porous PDMS with Negative Piezopermittivity
for Sensing and Energy Storage Applications. ACS Appl. Polym. Mater..

[26] Wang C. J., Kuan W. F., Lin H. P., Shchipunov Y. A., Chen L. J. (2021). Facile hydrophilic modification of polydimethylsiloxane-based
sponges for efficient oil-water separation. J. Ind. Eng. Chem..

[27] Shi J., Zhang H., Jackson J., Shademani A., Chiao M. (2016). A robust and refillable magnetic
sponge capsule for remotely triggered
drug release. J. Mater. Chem. B.

[28] Frost M. C., Meyerhoff M. E. (2004). Controlled
photoinitiated release of nitric oxide from
polymer films containing *S*-nitroso-*N*-acetyl-DL-penicillamine derivatized fumed silica filler. J. Am. Chem. Soc..

[29] Thurgood P., Baratchi S., Szydzik C., Mitchell A., Khoshmanesh K. (2017). Porous PDMS
structures for the storage and release of aqueous solutions into fluidic
environments. Lab Chip.

[30] Shi K., Aviles-Espinosa R., Rendon-Morales E., Woodbine L., Salvage J. P., Maniruzzaman M., Nokhodchi A. (2021). Magnetic Field Triggerable Macroporous
PDMS Sponge Loaded with an Anticancer Drug, 5-Fluorouracil. ACS Biomater. Sci. Eng..

[31] Ozkan E., Garren M., Manuel J., Douglass M., Devine R., Mondal A., Kumar A., Ashcraft M., Pandey R., Handa H. (2023). Superhydrophobic and
Conductive Foams with Antifouling and Oil-Water
Separation Properties. ACS Appl. Mater. Interfaces.

[32] Nikpour S., Ansari-Asl Z., Sedaghat T., Hoveizi E. (2022). Curcumin-loaded Fe-MOF/PDMS
porous scaffold: Fabrication, characterization, and biocompatibility
assessment. J. Ind. Eng. Chem..

[33] Lou X., Munro S., Wang S. (2004). Drug release
characteristics of phase
separation pHEMA sponge materials. Biomaterials.

[34] Bakshi S., Pandey K., Bose S., Gunjan, Paul D., Nayak R. (2019). Permanent
superhydrophilic surface modification in microporous polydimethylsiloxane
sponge for multi-functional applications. J.
Colloid Interface Sci..

[35] Ozkan E., Garren M., Manuel J., Douglass M., Devine R., Mondal A., Kumar A., Ashcraft M., Pandey R., Handa H. (2023). Superhydrophobic and
Conductive Foams with Antifouling and Oil–Water
Separation Properties. ACS Appl. Mater. Interfaces.

[36] Zhu D. Y., Handschuh-Wang S., Zhou X. C. (2017). Recent progress in fabrication and
application of polydimethylsiloxane sponges. J. Mater. Chem. A.

[37] Herren B., Webster V., Davidson E., Saha M. C., Altan M. C., Liu Y. T. (2021). PDMS Sponges with Embedded Carbon Nanotubes as Piezoresistive
Sensors for Human Motion Detection. Nanomaterials.

[38] Si J., Cui Z., Xie P., Song L., Wang Q., Liu Q., Liu C. (2016). Characterization of 3 D elastic porous polydimethylsiloxane
(PDMS)
cell scaffolds fabricated by VARTM and particle leaching. J. Appl. Polym. Sci..

[39] Zheng X. D., Ji B., Jiang R., Cui Y. W., Xu T. T., Zhou M., Li Z. Y. (2022). Polydimethylsiloxane/carbonized
bacterial cellulose sponge for oil/water
separation. Process Saf. Environ. Prot..

[40] Shin J. H., Heo J. H., Jeon S., Park J. H., Kim S., Kang H. W. (2019). Bio-inspired hollow
PDMS sponge for enhanced oil-water
separation. J. Hazard. Mater..

[41] Stern, S. A. ; Fried, J. R. Permeability of polymers to gases and vapors. In Physical Properties of Polymers Handbook; Springer, 2007; pp 1033–1047.

[42] Chug M. K., Feit C., Brisbois E. J. (2019). Increasing
the Lifetime of Insulin
Cannula with Antifouling and Nitric Oxide Releasing Properties. ACS Appl. Bio Mater..

[43] Chug M. K., Bachtiar E., Narwold N., Gall K., Brisbois E. J. (2021). Tailoring
nitric oxide release with additive manufacturing to create antimicrobial
surfaces. Biomater. Sci..

[44] Chug M. K., Brisbois E. J. (2022). Smartphone compatible nitric oxide releasing insert
to prevent catheter-associated infections. J.
Controlled Release.

[45] Gillis V. E., van Es M. J., Wouters Y., Wanten G. J. (2023). Antiseptic barrier
caps to prevent central line-associated bloodstream infections: A
systematic review and meta-analysis. Am. J.
Infect. Control.

[46] Brisbois E. J., Handa H., Major T. C., Bartlett R. H., Meyerhoff M. E. (2013). Long-term
nitric oxide release and elevated temperature stability with S-nitroso-N-acetylpenicillamine
(SNAP)-doped Elast-eon E2As polymer. Biomaterials.

[47] Chug M. K., Crutchfield N., Garren M., Handa H., Brisbois E. J. (2024). Engineering
Nitric Oxide-Releasing Antimicrobial Dental Coating for Targeted Gingival
Therapy. ACS Appl. Bio Mater..

[48] Cooper G. L., Schiller A. L., Hopkins C. C. (1988). Possible
Role of Capillary Action
in Pathogenesis of Experimental Catheter-Associated Dermal Tunnel
Infections. J. Clin. Microbiol..

[49] Jamal M., Ahmad W., Andleeb S., Jalil F., Imran M., Nawaz M. A., Hussain T., Ali M., Rafiq M., Kamil M. A. (2018). Bacterial biofilm and associated
infections. J. Chin. Med. Assoc..

[50] Roy H. (2009). Tuning the
Properties of the Bacterial Membrane with Aminoacylated Phosphatidylglycerol. IUBMB Life.

[51] Estes L. M., Singha P., Singh S., Sakthivel T. S., Garren M., Devine R., Brisbois E. J., Seal S., Handa H. (2021). Characterization of a nitric oxide (NO) donor molecule and cerium
oxide nanoparticle (CNP) interactions and their synergistic antimicrobial
potential for biomedical applications. J. Colloid
Interface Sci..

[52] Ullmann B. D., Myers H., Chiranand W., Lazzell A. L., Zhao Q., Vega L. A., Lopez-Ribot J. L., Gardner P. R., Gustin M. C. (2004). Inducible
defense mechanism against nitric oxide in. Eukaryotic
Cell.

[53] Johnson C. J., Eix E. F., Lam B. C., Wartman K. M., Meudt J. J., Shanmuganayagam D., Nett J. E. (2021). Augmenting the Activity of Chlorhexidine
for Decolonization of *Candida auris* from Porcine
skin. J. Fungi.

[54] Sahiner A., Halat E., Algin Yapar E. (2019). Comparison of bactericidal and fungicidal
efficacy of antiseptic formulations according to EN 13727 and EN 13624
standards. Turk. J. Med. Sci..

[55] Garrett T. R., Bhakoo M., Zhang Z. B. (2008). Bacterial
adhesion and biofilms on
surfaces. Prog. Nat. Sci.: Mater. Int..

[56] Khan H. A., Ahmad A., Mehboob R. (2015). Nosocomial
infections and their control
strategies. Asian Pac. J. Trop. Biomed..

[57] Kojic E. M., Darouiche R. O. (2004). Infections
of medical devices. Clin. Microbiol. Rev..

[58] Goodman A. B., Chug M. K., Crutchfield N., Handa H., Brisbois E. J. (2025). Nitric
Oxide-Releasing Polydimethylsiloxane Sponges with Tunable Porosity. ACS Appl. Mater. Interfaces.

[59] Xu L. C., Wo Y., Meyerhoff M. E., Siedlecki C. A. (2017). Inhibition of bacterial adhesion
and biofilm formation by dual functional textured and nitric oxide
releasing surfaces. Acta Biomater..

[60] Ashcraft M., Douglass M., Garren M., Mondal A., Bright L. E., Wu Y., Handa H. (2022). Nitric Oxide-Releasing
Lock Solution for the Prevention
of Catheter-Related Infection and Thrombosis. ACS Appl. Bio Mater..

[61] Homeyer K. H., Goudie M. J., Singha P., Handa H. (2019). Liquid-Infused Nitric-Oxide-Releasing
Silicone Foley Urinary Catheters for Prevention of Catheter-Associated
Urinary Tract Infections. ACS Biomater. Sci.
Eng..

[62] Mondal A., Singha P., Douglass M., Estes L., Garren M., Griffin L., Kumar A., Handa H. (2021). A Synergistic New Approach
Toward Enhanced Antibacterial Efficacy via Antimicrobial Peptide Immobilization
on a Nitric Oxide-Releasing Surface. ACS Appl.
Mater. Interfaces.

[63] Devine R., Douglass M., Ashcraft M., Tayag N., Handa H. (2021). Development
of Novel Amphotericin B-Immobilized Nitric Oxide-Releasing Platform
for the Prevention of Broad-Spectrum Infections and Thrombosis. ACS Appl. Mater. Interfaces.

[64] Akbıyık A., Kaya S., Aksun M. (2023). Determination of microbial
contamination
on the outer surface of needleless connectors before and after disinfection. Intensive Crit. Care Nurs..

[65] Flynn J. M., Larsen E. N., Keogh S., Ullman A. J., Rickard C. M. (2019). Methods
for microbial needleless connector decontamination: a systematic review
and meta-analysis. Am. J. Infect. Control.

[66] Donlan R. M. (2001). Biofilms
and device-associated infections. Emerging Infect.
Dis..

[67] Nandakumar V., Chittaranjan S., Kurian V. M., Doble M. (2013). Characteristics of
bacterial biofilm associated with implant material in clinical practice. Polym. J..

[68] Veerachamy S., Yarlagadda T., Manivasagam G., Yarlagadda P. K. (2014). Bacterial
adherence and biofilm formation on medical implants: a review. Proc. Inst. Mech. Eng., Part H.

[69] Moorer W. (2003). Antiviral
activity of alcohol for surface disinfection. Int. J. Dent. Hyg..

[70] Tarasenko O., Scott A., Soderberg L., Ponnappan U., Alusta P. (2010). Killing of Bacillus spores is mediated
by nitric oxide
and nitric oxide synthase during glycoconjugate-enhanced phagocytosis. Glycoconjugate J..

[71] Lazar E. E., Wills R. B. H., Ho B. T., Harris A. M., Spohr L. J. (2008). Antifungal
effect of gaseous nitric oxide on mycelium growth, sporulation and
spore germination of the postharvest horticulture pathogens, *Aspergillus niger, Monilinia fructicola* and *Penicillium
italicum*. Lett. Appl. Microbiol..

[72] Jenkins D. R. (2017). Nosocomial
infections and infection control. Medicine.

