# Supplementary material for: Dual Action Nitric Oxide-Releasing Polydimethylsiloxane Sponge: Preventing Infection in Needleless Connectors
Source: ACS Appl Bio Mater. 2025 Sep 12;8(10):8925–39. doi: 10.1021/acsabm.5c01100 (PMC12541702; doi:10.1021/acsabm.5c01100)
Supplement: Supplementary file 1 [file mt5c01100_si_001.pdf]

Supporting Information

Dual Action Nitric Oxide-Releasing Polydimethylsiloxane Sponge: Preventing Infection in Needleless Connectors

Adam Brooks Goodman[1†], Manjyot Kaur Chug[1†], Natalie Crutchfield[1], Mark Garren[1], Hitesh Handa[1,2], Elizabeth J. Brisbois*[1]

[†] Authors declare equal contributions

[1] School of Chemical, Materials, and Biomedical Engineering, College of Engineering, University of Georgia, Athens, Georgia, USA

[2] Department of Pharmaceutical and Biomedical Sciences, College of Pharmacy, University of Georgia, Athens, Georgia, USA

Corresponding Author:

Dr. Elizabeth J. Brisbois

Associate Professor

School of Chemical, Materials, and Biomedical Engineering

University of Georgia

302 East Campus Rd

Athens, GA 30602

Telephone: 706-542-1243

E-mail: ejbrisbois@uga.edu

1. Supplementary Methods

S1.1 Nitric Oxide Release Using a Gold-Standard Nitric Oxide Analyzer

To ensure nitric oxide (NO) was releasing from the SNAP loaded sponges, a chemiluminescence Nitric Oxide Analyzer (NOA 280i, Sievers, Boulder, CO) was used to measure the instantaneous release kinetics. As IPA evaporates quickly, the vapors pose a threat to the NOA. Therefore, NO release from the 82% porous sponge was examined as a representative. Briefly, SNAP-IPA swelled sponge samples were immediately weighed then wrapped in Tegaderm™ to reduce the risk of IPA evaporation while allowing for NO to permeate out and into the reaction chamber.

Samples were placed into an amber cell chamber after a baseline measurement was reached at 37 °C and NO was continuously purged from the samples. Nitric oxide released from the sponges reacted with ozone in an internal reaction chamber, releasing a photon with a light intensity proportional to the quantity of NO released. The NO flux was determined using the instantaneous NO release, expressed in parts per billion (ppb), and normalized to the samples weight (x 10^{-10} mol mg^{-1} min^{-1}).

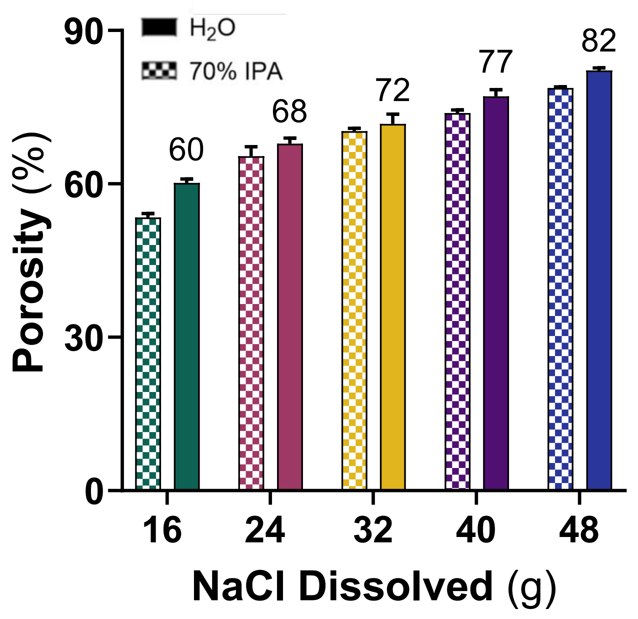

Figure S1. Porosity comparison of PDMS sponges with PEO reveals similar trends with DI water and 70% IPA. Average porosity values (%) for each sponge type with DI water are shown on top of corresponding PDMS-PEO bars. Data represents the mean ± standard deviation (*n = 3*).

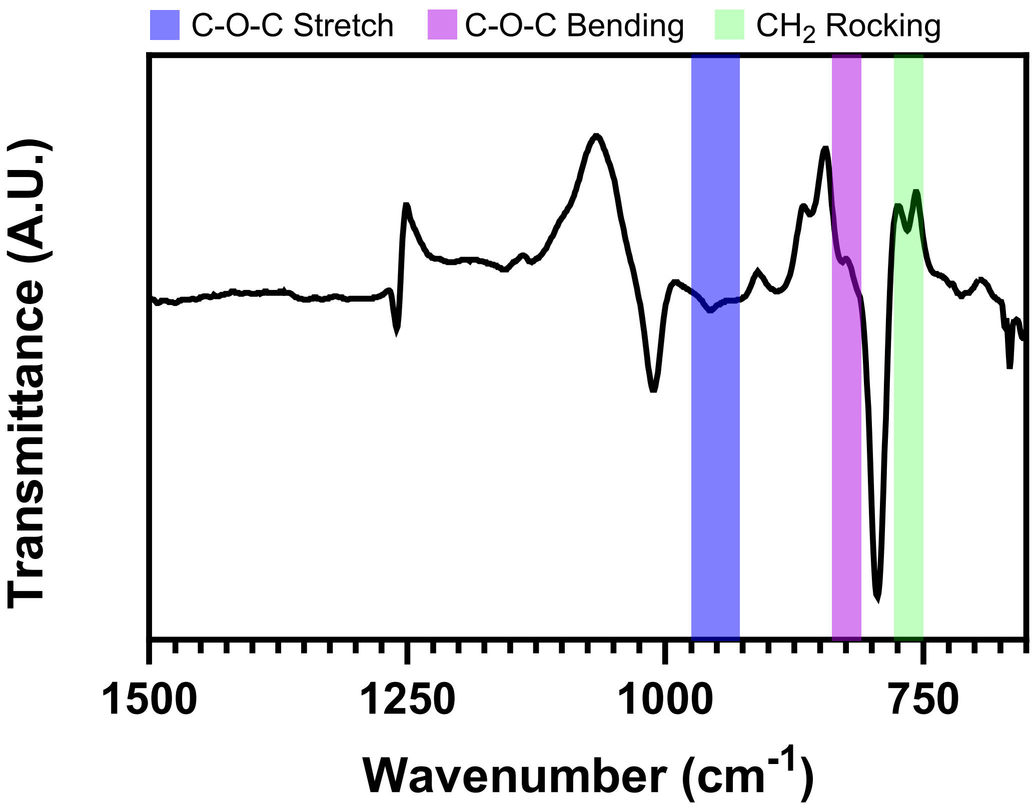

Figure S2. Representative UATR-FTIR subtraction spectra of 82% porous PDMS and PDMS-*b*-PEO sponges. Peaks at 965cm^{-1} can be assigned to CH_2 rocking and C-O-C stretch, while peaks at 857cm^{-1} correspond to CH_2 rocking and C-O-C bending, and peaks at 767cm^{-1} correspond to CH_2 rocking.[1]

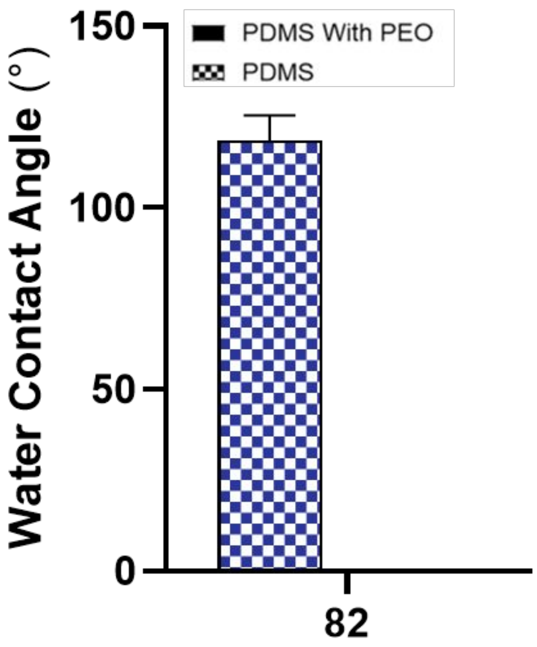

Figure S3. Static water contact angle measurements of PDMS sponges with and without PEO. A 10 µL droplet of deionized water was applied at multiple locations on each sponge surface Data represents the mean ± standard deviation (*n = 4*).

Video S1. Water droplet absorption on the surface of the hydrophilic-modified 82% porous PDMS sponge, demonstrating rapid absorption at multiple points. This highlights the successful incorporation of the surfactant into the sponge material post-fabrication.

Video S1 Still:

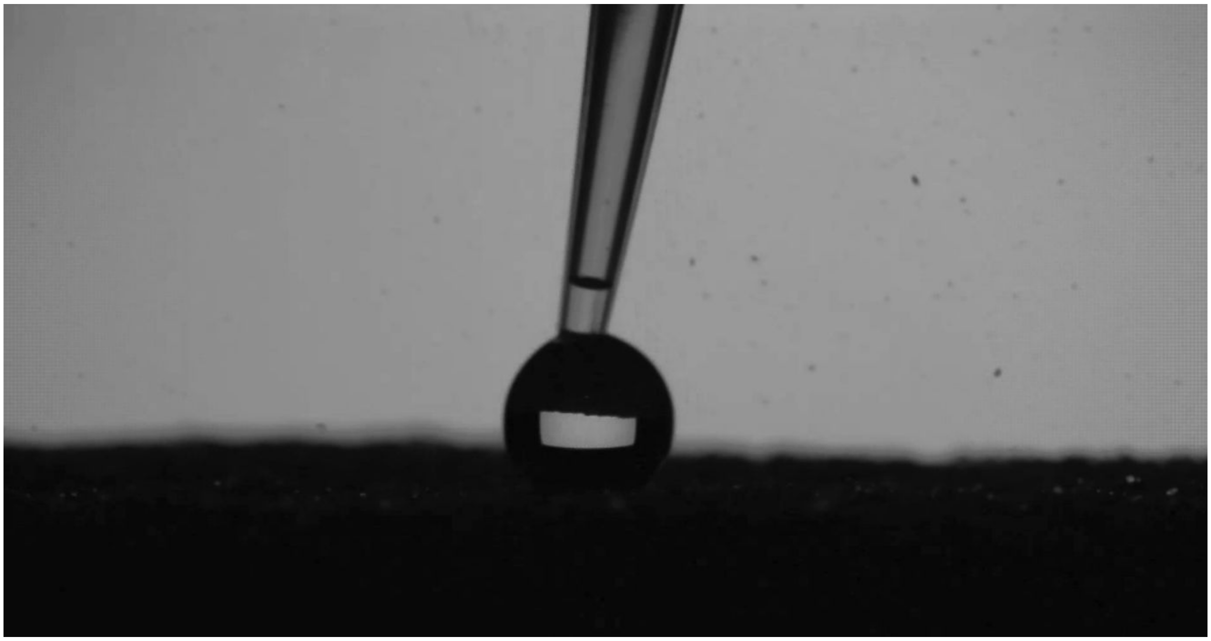

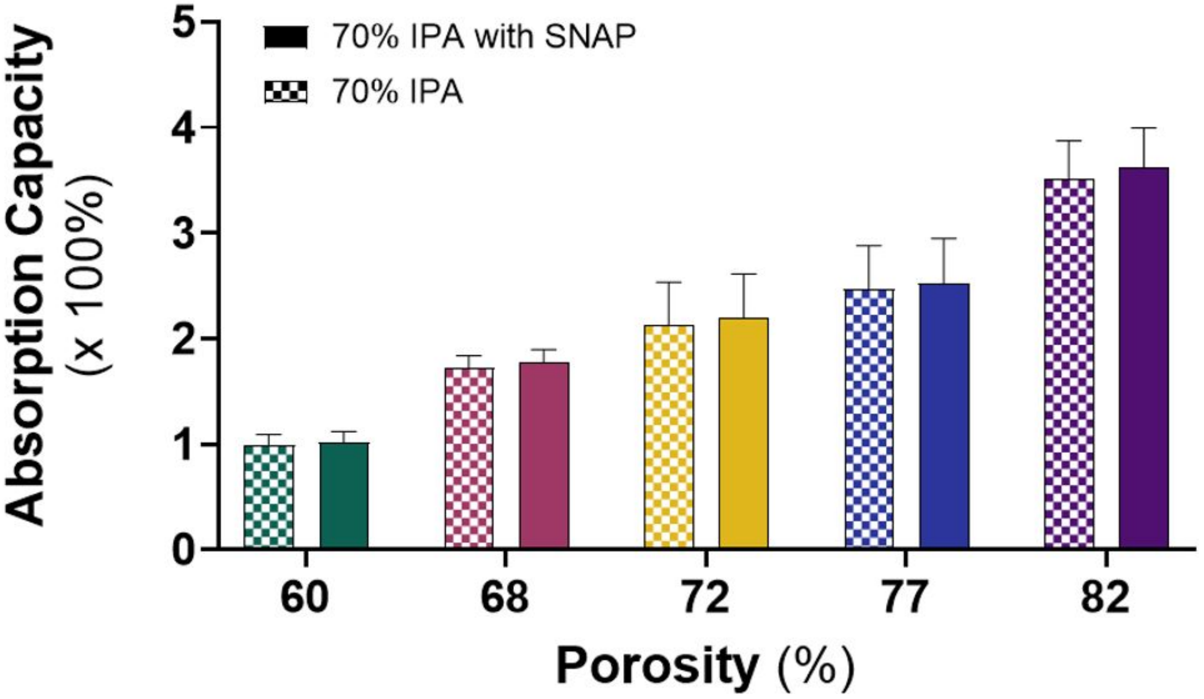

Figure S4. Absorption capacity of PDMS-*b*-PEO sponges with and without SNAP after 15 min. Addition of SNAP to the 70% IPA solution shows no significant difference in absorption capacity. Data represents the mean ± standard deviation (*n = 3*).

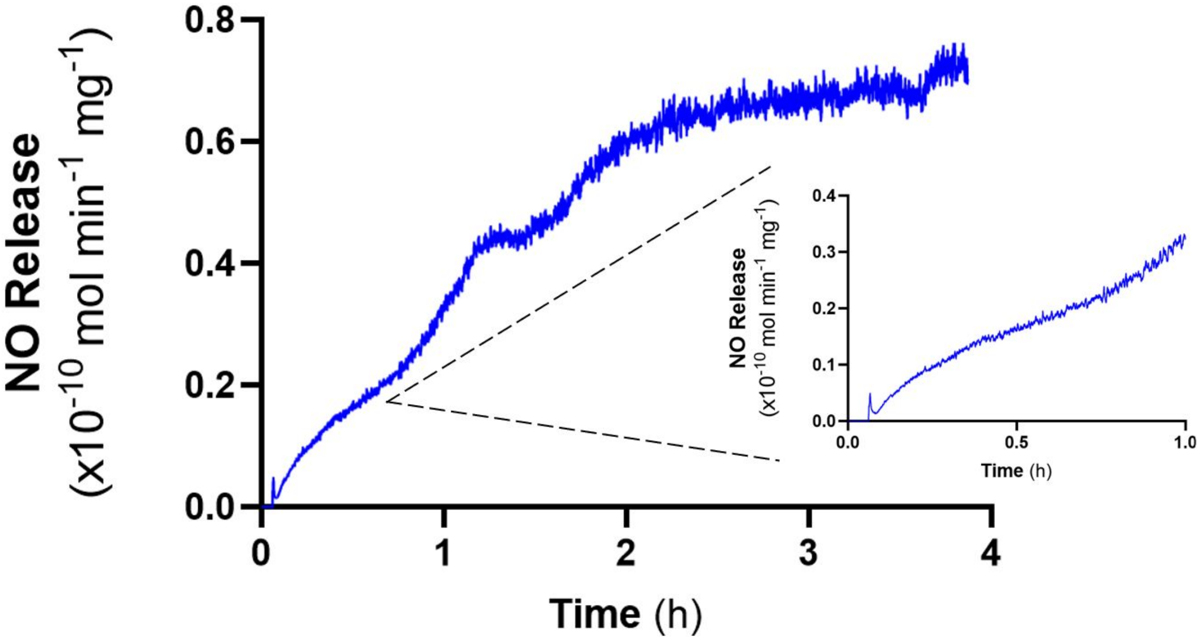

Figure S5. Representative nitric oxide release profile of the 82% porous sponge swollen in SNAP-IPA solution using an NOA following methods from **S1.1**.

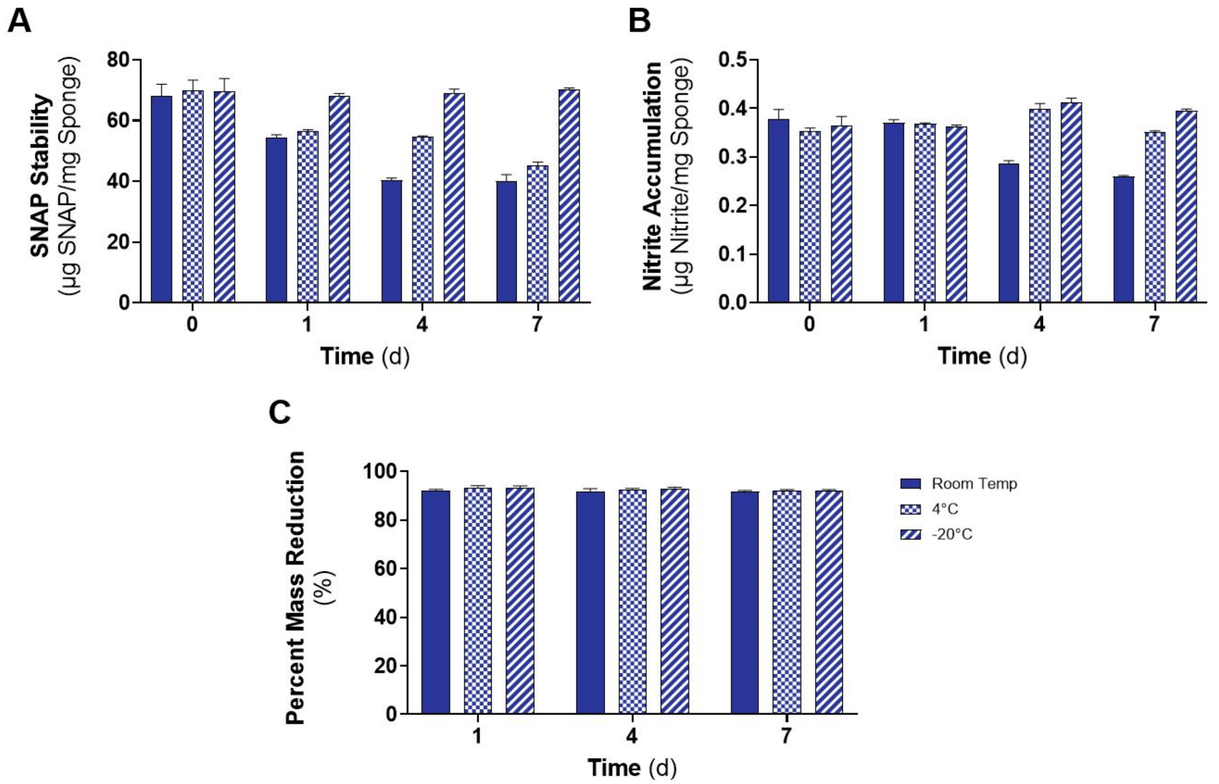

Figure S6. Storage stability of 82% porous SNAP-IPA sponges stored at room temperature, 4 °C, or -20 °C for 7 d. **(A)** Stability of SNAP after storage using 24 h leachates in PBS. **(B)** Nitrite production of 24 h sponge leachates in PBS. **(C)** Percent mass loss of sponges after storage. Data represents the mean ± standard deviation (*n* = *3*).

Table S1: Representative average pore size distribution of PDMS sponges with and without the PDMS-*b*-PEO surfactant (PEO). Data represents the mean ± standard deviation (*n* = *1*) from 30 sites.

PDMS Sponge Porosity (%)	PDMS-PEO Sponge Porosity (%)	Pore Size (µm) PDMS Sponge	Pore Size (µm) PDMS-PEO Sponge
59.6 ± 1.48	60.2 ± 0.77	248.1 ± 108.9	216.4 ± 80.94
66.5 ± 0.07	67.9 ± 1.09	261.8 ± 97.82	246.1 ± 95.45
69.6 ± 0.61	71.7 ± 1.88	254.7 ± 118.8	280.8 ± 106.1
76.9 ± 2.03	77.1 ± 1.33	228.3 ± 85.48	234.9 ± 75.81
82.7 ± 0.22	82.2 ± 0.49	265.3 ± 104.6	237.9 ± 74.68

Table S2. Zone of inhibition diameter measurements for each sponge formulation on various microbes. Data represents the mean ± standard deviation (*n* ≥ *3*).

Zone of Inhibition (cm)										
**Sponge Porosity** (%)	***E. coli*** **ATCC 25922**		***P. aeruginosa*** **ATCC 27853**		***S. aureus*** **ATCC 6538**		***S. epidermidis*** **ATCC 35984**		***C. albicans*** **ATCC MYA4441**	
	70% IPA	SNAP-70% IPA	70% IPA	SNAP-70% IPA	70% IPA	SNAP-70% IPA	70% IPA	SNAP-70% IPA	70% IPA	SNAP-70% IPA
**68**	0.63 ± 0.20	1.17 ± 0.08	0.59 ± 0.03	0.79 ± 0.06	0.59 ± 0.05	1.40 ± 0.07	0.59 ± 0.08	2.91 ± 0.24	0.63 ± 0.09	0.63 ± 0.05
**72**	0.70 ± 0.04	1.33 ± 0.10	0.58 ± 0.10	1.00 ± 0.01	0.50 ± 0.05	1.68 ± 0.03	0.63 ± 0.06	3.14 ± 0.17	0.58 ± 0.07	0.81 ± 0.08
**77**	0.88 ± 0.03	1.52 ± 0.12	0.69 ± 0.08	1.03 ± 0.12	0.56 ± 0.05	1.68 ± 0.05	0.65 ± 0.06	3.46 ± 0.08	0.61 ± 0.03	0.89 ± 0.08
**82**	0.95 ± 0.06	1.66 ± 0.19	0.58 ± 0.06	1.20 ± 0.01	0.60 ± 0.09	2.12 ± 0.18	0.69 ± 0.03	3.61 ± 0.10	0.65 ± 0.04	1.45 ± 0.15

Table S3: Log_{10} reductions in viable planktonic microbes exposed to 82% porous antimicrobial sponges for 4 h compared to control PDMS sponges. Data represents the mean ± standard deviation (*n* = *4*).

Planktonic Log_{10} Reduction Values for 82% Antimicrobial Sponges					
E. coli **ATCC 25922**		***S. aureus*** **ATCC 6538**		***C. albicans*** **ATCC MYA4441**	
70% IPA	SNAP-70% IPA	70% IPA	SNAP-70% IPA	70% IPA	SNAP-70% IPA
0.12 ± 0.11	0.53 ± 0.14	0.37 ± 0.17	3.02 ± 0.11	0.02 ± 0.08	0.67 ± 0.31

Table S4: Log_{10} reductions in viable microbes adhered to luer connectors exposed to 82% porous IPA or SNAP-IPA sponges for 30 min compared to control microbial-adhered luer connectors. Data represents the mean ± standard deviation ($n \geq 4$).

Adhered Log_{10} Reduction Values for 82% Porous Sponges									
E. coli **ATCC 25922**		***P. aeruginosa*** **ATCC 27853**		***S. aureus*** **ATCC 6538**		***S. epidermidis*** **ATCC 35984**		***C. albicans*** **ATCC MYA4441**	
70% IPA	SNAP-70% IPA	70% IPA	SNAP-70% IPA	70% IPA	SNAP-70% IPA	70% IPA	SNAP-70% IPA	70% IPA	SNAP-70% IPA
2.64 ± 0.24	3.50 ± 0.98	4.04 ± 0.44	7.01 ± 0.15	1.61 ± 0.36	2.47 ± 0.78	2.97 ± 0.71	4.53 ± 1.60	4.18 ± 0.86	5.62 ± 0.16

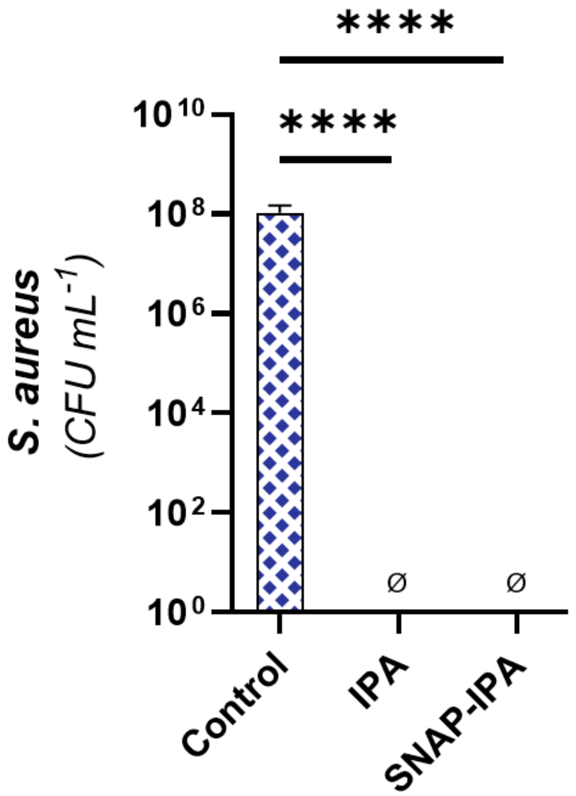

Figure S7. *S. aureus* viability of contaminated luer connectors after 24 h exposure to IPA and SNAP-IPA 82% porous sponges. Data represents the mean ± standard deviation ($n = 4$). * Indicates significance: **** ($p<0.0001$). Ø indicates results were below the detection limit.

References:

(1) Parameswaran, C., Chaudhary, R. P., Prutvi, S. H., Gupta, D. Rapid One Step Fabrication of Hydrophilic Hierarchical Porous PDMS with Negative Piezopermittivity for Sensing and Energy Storage Applications. *ACS Appl. Polym. Mater.* **2022**, *4* (3), 2047-2056. 10.1021/acsapm.1c00593
